# MTH1 and OGG1 maintain a low level of 8-oxoguanine in Alzheimer's brain, and prevent the progression of Alzheimer's pathogenesis

**DOI:** 10.1038/s41598-021-84640-9

**Published:** 2021-03-23

**Authors:** Sugako Oka, Julio Leon, Kunihiko Sakumi, Nona Abolhassani, Zijing Sheng, Daisuke Tsuchimoto, Frank M. LaFerla, Yusaku Nakabeppu

**Affiliations:** 1 Division of Neurofunctional Genomics, Department of Immunobiology and Neuroscience, Medical Institute of Bioregulation, Kyushu University, Fukuoka, 812-8582 Japan; 2 Department of Neurobiology and Behavior, University of California, Irvine, CA 92697 USA; 3 Present Address: Department of Cancer Biology, University of Kansas Medical Center, Kansas City, KS 66010 USA; 4 Present Address: Laboratory for Advanced Genomics Circuit, RIKEN Center for Integrative Medical Sciences, Yokohama, 230-0045 Japan

**Keywords:** Cell death in the nervous system, Alzheimer's disease

## Abstract

8-Oxoguanine (8-oxoG), a major oxidative base lesion, is highly accumulated in Alzheimer’s disease (AD) brains during the pathogenic process. MTH1 hydrolyzes 8-oxo-dGTP to 8-oxo-dGMP, thereby avoiding 8-oxo-dG incorporation into DNA. 8-OxoG DNA glycosylase-1 (OGG1) excises 8-oxoG paired with cytosine in DNA, thereby minimizing 8-oxoG accumulation in DNA. Levels of MTH1 and OGG1 are significantly reduced in the brains of sporadic AD cases. To understand how 8-oxoG accumulation in the genome is involved in AD pathogenesis, we established an AD mouse model with knockout of *Mth1* and *Ogg1* genes in a 3xTg-AD background. MTH1 and OGG1 deficiency increased 8-oxoG accumulation in nuclear and, to a lesser extent, mitochondrial genomes, causing microglial activation and neuronal loss with impaired cognitive function at 4–5 months of age. Furthermore, minocycline, which inhibits microglial activation and reduces neuroinflammation, markedly decreased the nuclear accumulation of 8-oxoG in microglia, and inhibited microgliosis and neuronal loss. Gene expression profiling revealed that MTH1 and OGG1 efficiently suppress progression of AD by inducing various protective genes against AD pathogenesis initiated by Aß/Tau accumulation in 3xTg-AD brain. Our findings indicate that efficient suppression of 8-oxoG accumulation in brain genomes is a new approach for prevention and treatment of AD.

## Introduction

Oxidative damage to DNA can cause mutagenesis and programmed cell death; the former may result in carcinogenesis, while the latter often causes degenerative disorders^1–3^. Oxidative stress is associated with the age-related neurodegenerative disorder, Alzheimer’s disease (AD)^4,5^. AD, the most common cause of dementia, is pathologically characterized by amyloid plaques, neurofibrillary tangles, and neuronal loss or neurodegeneration ^6^. Accumulation of amyloid β (Aβ) in the cytoplasm induces mitochondrial dysfunction and the production of reactive oxygen species (ROS)^7,8^. Increased ROS generation from aged mitochondria accelerates Aβ accumulation via increased amyloidogenic APP processing^9^. Thus, the vicious cycle of ROS and Aβ-accumulation has a pivotal role in the development of AD^10,11^.

Among the various types of oxidative damage, 8-oxo-7,8-dihydroguanine, also known as 8-oxoguanine (8-oxoG), an oxidized form of guanine, is one of the major oxidized base lesions in nucleotide pools, DNA and RNA^12–15^. 8-OxoG in DNA is highly mutagenic and cytotoxic because it can pair with adenine as well as cytosine; therefore, mammalian cells are equipped with two distinct enzymes to minimize 8-oxoG accumulation in DNA. MTH1, also known as NUDT1, hydrolyzes 8-oxo-7,8-dihydro-2´-deoxyguanosine-5´-triphosphate (8-oxo-dGTP) to 8-oxo-dGMP, thereby avoiding incorporation of 8-oxo-dGTP into DNA ^1^. 8-OxoG DNA glycosylase-1 (OGG1) excises 8-oxoG paired with cytosine in DNA^16^, thereby minimizing the accumulation of 8-oxoG in DNA^3^.

8-OxoG becomes highly accumulated in AD patient brains^17–20^. Although sex-specific and genetic risk factors were not excluded from the findings of these previous studies, 8-oxoG levels were significantly higher compared with age‐matched control subjects in a range of brain regions, including the frontal, temporal, and parietal lobes, and in the ventricular cerebrospinal fluid. These studies used various methods, such as immunostaining or gas chromatography/mass spectrometry, and their results suggest that 8-oxoG accumulation in the brain may be involved in the development or progression of AD. We have demonstrated that levels of MTH1 and OGG1 are significantly decreased in sporadic AD brains^21,22^. Moreover, *OGG1* mutations that cause the loss or reduction of OGG1 capacity have been found in AD patients^23^. Because MTH1 and/or OGG1-deficient mice are vulnerable to neurodegeneration under increased oxidative stress^13,24–27^, we hypothesized that a reduced capacity to minimize 8-oxoG accumulation in DNA and increased 8-oxoG accumulation in brain DNA are causatively associated with AD^3,28^.

## Results

### *Mth1/Ogg1* knockout in 3xTg-AD-H mice induces neurodegeneration accompanied with Aβ accumulation in the brain, and exacerbates cognitive impairment

To investigate the role of 8-oxoG accumulated in DNA during the development of AD, we introduced *Mth1/Ogg1* null alleles into homozygous triple-transgenic AD model mice (3xTg-AD-H), which carry homozygous *APP*_Swe_ and *MAPT*_P301L_ transgenes and a homozygous *Psen1*_M146V_-knockin mutation^29^. We established three lines of mice: NT, carrying no transgene or mutant allele; ADH/WT, carrying homozygous *APP*_Swe_/*MAPT*_P301L_ transgenes and the *Psen1*_M146V_ mutation with homozygous *Mth1*/*Ogg1* wild-type alleles; and ADH/TO-DKH, carrying homozygous *APP*_Swe_/*MAPT*_P301L_ transgenes and the *Psen1*_M146V_ mutation with homozygous *Mth1*/*Ogg1* null alleles. *Mth1* and *Ogg1* null alleles were confirmed by genomic PCR (Supplementary Fig. S1).

**Figure 1:**
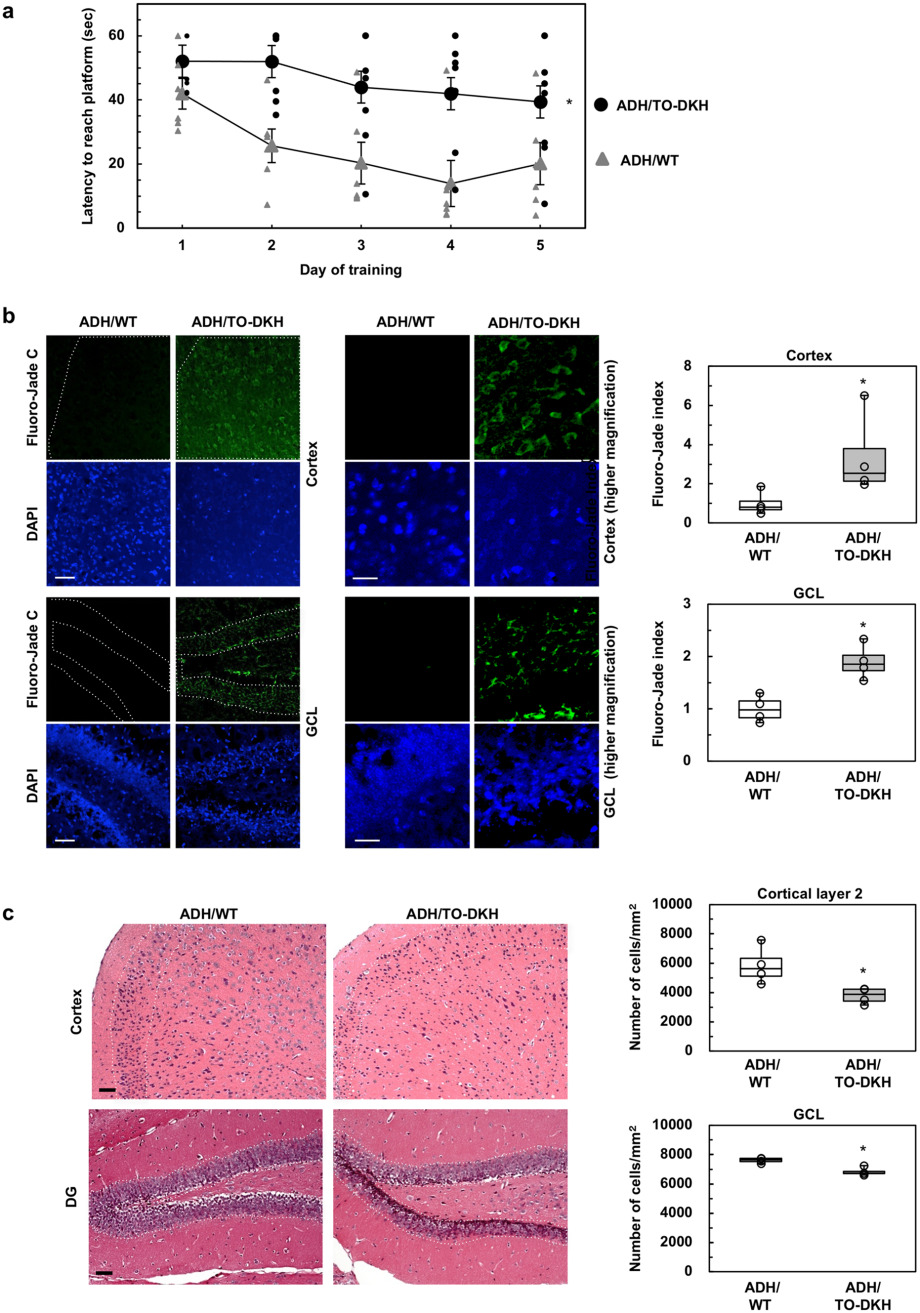
*Mth1*/*Ogg1* knockout exacerbates cognitive impairment and triggers neurodegeneration. (**a**) MWM test (non-cued test). Latencies to reach the platform are shown. *n* = 6–8. Data are shown as the mean ± SEM. MANOVA: genotype, *p = 0.0024; day, *p* = 0.0037. (**b**) Detection of neurodegeneration in the cortex and GCL using Fluoro-Jade C staining. Nuclei are stained with DAPI. Scale bar = 50 µm. Higher magnification images of ADH/TO-DKH are shown in the right. Scale bar = 20 µm. Fluoro-Jade C fluorescence intensity in the region enclosed by the dotted line was measured and the intensity per unit area was defined as Fluoro-Jade index, and is shown relative to that in ADH/WT. *n* = 4. Wilcoxon exact test (two-sided), *p = 0.0286. (**c**) Hematoxylin and Eosin staining. Scale bar = 50 µm. Number of hematoxylin-stained nuclei in the region enclosed by the dotted line was counted in each digital image and the number per unit area was defined. *n* = 4. Wilcoxon exact test (two-sided), *p = 0.0286.

3xTg-AD-H mice exhibit a decline in spatial working memory in Morris water maze (MWM) tests, as early as 6 months of age^30^. Therefore, to evaluate whether *Mth1*/*Ogg1* knockout accelerates AD phenotype, we used 3xTg-AD-H mice at 4–5 months of age before the appearance of learning disability or pathological alteration of AD. We assessed the three lines of mice, NT, ADH/WT, and ADH/TO-DKH in MWM tests. Compared with NT, ADH/WT mice had essentially the same performance during 5-day training (Supplementary Fig. S2). However, ADH/TO-DKH mice exhibited severely impaired spatial working memory testing in the MWM compared with ADH/WT (Fig. 1a).

We next examined whether *Mth1*/*Ogg1* knockout induces neuronal loss or neurodegeneration in the ADH/WT brain at 4–5 months of age. Brain sections were stained with Fluoro-Jade C, which reacts with degenerating neuronal cell bodies, distal dendrites, axons, and terminals^31^. In ADH/WT brain, green fluorescence derived from bound Fluoro-Jade C was barely detectable, giving no evidence of degenerating neurons (Fig. 1b). In contrast, ADH/TO-DKH brains exhibited significantly higher green fluorescence in the cortex, especially in layers 4 and 5 (Fig. 1b, upper panels), and a weaker signal in the granule cell layer (GCL) of the dentate gyrus (DG) and hilus (Fig. 1b, lower panels). As shown in Supplementary Fig. S3, green fluorescence was mostly observed in NeuN-positive neurons in the ADH/TO-DKH cortex.

**Figure 2:**
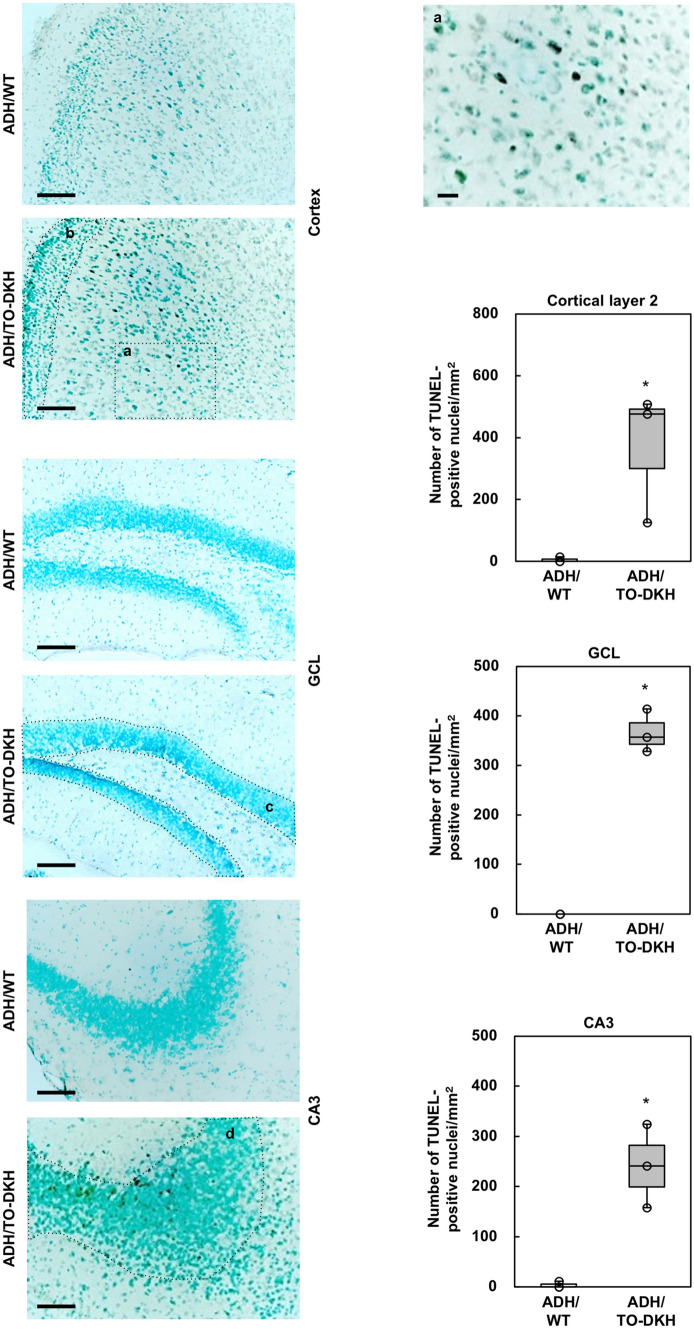
Detection of TUNEL-positive cells in the ADH/TO-DKH brain. TUNEL-positive nuclei indicated by the black signals were observed in the cortex, dentate gyrus and CA3 subregion of the hippocampus of ADH/TO-DKH but not ADH/WT brains. Nuclei were counterstained with methyl green. Scale bar = 100 μm. The top-right panel (**a**) shows a magnified image of the boxed region (**a**) in the cortex of the ADH/TO-DKH brain. Scale bar = 20 μm. The numbers of TUNEL-positive nuclei in cortical layer 2, the GCL and CA3 areas outlined by the dotted lines in (**b**), (**c**) and (**d**) were counted and are shown in the graphs. n = 3. Wilcoxon exact test (one-sided), *p = 0.05.

Fluoro-Jade C staining suggested that neuronal damage in ADH/TO-DKH brains is evident in the cortex, and to a lesser extent in the GCL of the DG; therefore, we counted the number of hematoxylin-stained nuclei in hematoxylin/eosin-stained sections. Decreased cell density was evident in cortical layers 2–5 and in the GCL of the DG in ADH/TO-DKH compared with ADH/WT brains (Fig. 1c). The decrease in cell density was highly significant in cortical layer 2 (Fig. 1c, upper bar graph). In ADH/TO-DKH brains, Fluoro-Jade C fluorescence was weaker in cortical layer 2, probably because damaged neurons were already eliminated. We also detected endonucleolytic cleavage of nDNA, a characteristic of apoptosis, by TUNEL assay, and confirmed an increase in the number of TUNEL-positive nuclei in the cortex, GCL of the DG and CA3 in ADH/TO-DKH brains (Fig. 2).

**Figure 3:**
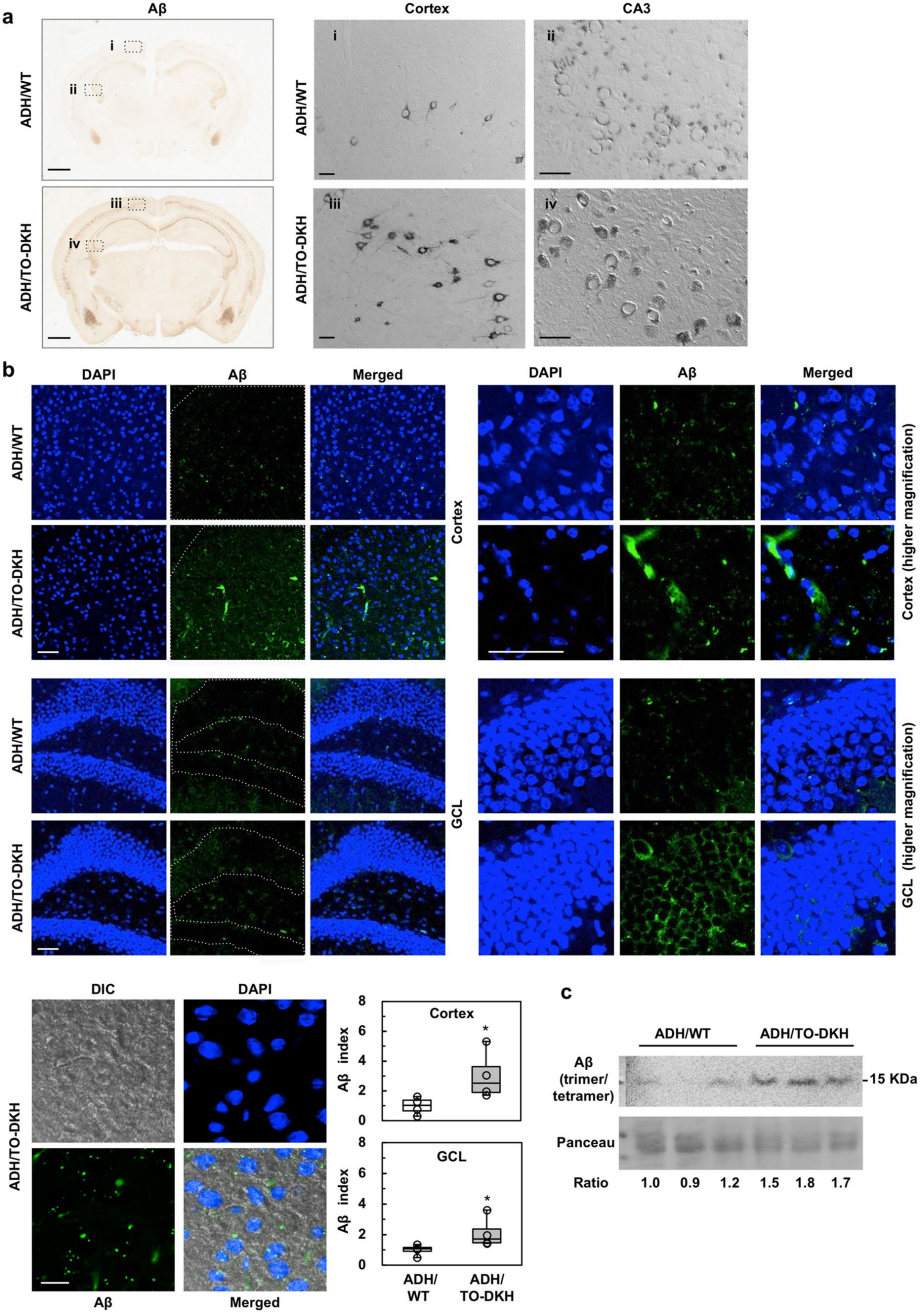
*Mth1*/*Ogg1* knockout accelerates AD pathophysiology. (**a**) Immunohistochemical detection of Aβ. Aβ levels are increased in ADH/TO-DKH compared with ADH/WT (left panels). Scale bar = 1 mm. DIC images of the boxed regions (i–iv) are shown in right panels. Cytoplasmic Aβ was more evident in the cortex, CA3 regions in ADH/TO-DKH. Scale bar = 50 µm. (**b**) Immunofluorescent detection of Aβ in the cortex and the granule cell layer (GCL) of the DG in ADH/WT and ADH/TO-DKH. Magnified images are shown in right panels. Scale bar = 50 µm. Magnified DIC images of the ADH/TO-DKH cortex are shown in left bottom panels. Scale bar = 20 µm. Aβ index in the region enclosed by the dotted line was measured. Aβ indices are shown as box plots. *n* = 4. Wilcoxon exact test (two-sided), *p = 0.0286. (**c**) Aβ accumulation in the ADH/TO-DKH cortex detected by western blotting analysis. Each sample was extracted from three individual mice, different from those used for immunostaining. The band of 15 KDa corresponds to Aβ (trimer/tetramer) in the SDS-soluble fraction. No band was detected in the SDS-insoluble/FA-extractable fraction. Ponceau staining was used as a loading control. Full-length gels are presented in Supplementary Figure S10. The relative ratio of the Aβ band (normalized by Ponceau staining) is shown. Student’s *t*-test, *p = 0.0071.

Next, we confirmed whether *Mth1*/*Ogg1* knockout accelerates AD pathology. Immunohistochemical detection of Aβ using an Aβ-N-terminal specific monoclonal antibody revealed that Aβ immunoreactivity (IR) was increased in the ADH/TO-DKH brain, especially in cortical layers 4–5 and in hippocampal CA1, CA2 and CA3 regions compared with ADH/WT brain. Aβ plaques were not, however, apparent (Fig. 3a, i–iv). Immunofluorescence microscopy also showed that levels of intracellular Aβ were significantly increased in the ADH/TO-DKH cortex and GCL of DG compared with the ADH/WT brain (Fig. 3b). Western blotting analysis of brain extracts revealed that levels of the 15 kDa band, corresponding to the Aβ trimer/tetramer, were higher in the ADH/TO-DKH cortex than in the ADH/WT cortex (Fig. 3c). These results indicate that *Mth1*/*Ogg1* knockout results in exacerbated AD pathophysiology in ADH/TO-DKH mice.

### *Mth1*/*Ogg1* knockout in 3xTg-AD-H mice increases accumulation of 8-oxoG in microglial nuclear DNA and causes microglial activation

**Figure 4:**
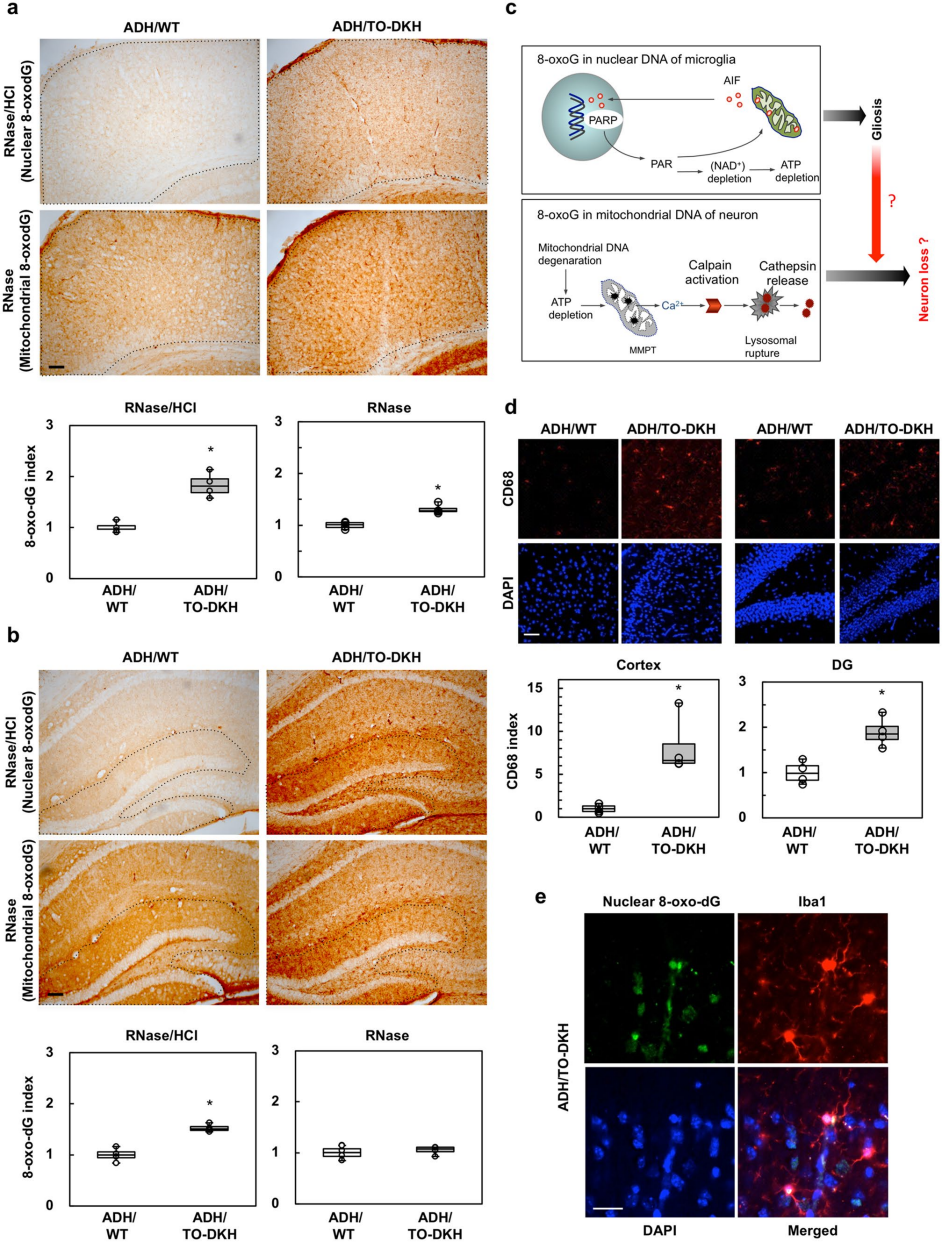
*Mth1*/*Ogg1* knockout in 3xTg-AD-H mice increases accumulation of 8-oxoG in microglial nuclear DNA and causes microglial activation. (**a**) Immunohistochemical detection of 8-oxo-dG in nuclei or mitochondria in the cortex of 4–5-month-old ADH/WT and ADH/TO-DKH mice. RNase/HCl: RNase-treated sections were further pretreated with 2 N HCl to detect 8-oxo-dG in nDNA. RNase: sections were pretreated with RNase only to detect 8-oxo-dG in mtDNA. Scale bar = 100 µm. Graphs show the relative 8-oxo-dG IRs (8-oxo-dG index) in the region enclosed by the dotted line. RNase/HCl (*n* = 4): Wilcoxon exact test (two-sided), *p = 0.0286. RNase (*n* = 4): Wilcoxon exact test (two-sided), *p = 0.0286. (**b**) Immunohistochemical detection of 8-oxo-dG in nuclei and mitochondria in the DG of ADH/WT and ADH/TO-DKH mice. Scale bar = 100 µm. Graphs show the relative 8-oxo-dG index in the nuclei (RNase/HCl), and mitochondria (RNase) in the DG. RNase/HCl (n = 4): Wilcoxon exact test (two-sided), *p = 0.0286. RNase (*n* = 4): Wilcoxon exact test (two-sided), *p* = 0.2. (**c**) Damaged neurons accumulate higher levels of 8-oxoG in mtDNA than in nDNA under oxidative conditions in the brain, while activated microglia mainly accumulate 8-oxoG in nuclear DNA. The former causes mitochondrial dysfunction and calpain-dependent neuronal dysfunction, and the latter results in poly (ADP-ribose) polymerase (PARP)-dependent activation of apoptosis-inducing factor (AIF) and exacerbated microgliosis. (**d**) Immunodetection of CD68. Scale bar = 50 µm. Graphs show the CD68 index. *n* = 4. Wilcoxon exact test (two-sided), *p = 0.0286. (**e**) Nuclear 8-oxo-dG was mostly detected in Iba1-positive microglia in the ADH/TO-DKH cortex. Scale bar = 20 µm.

8-OxoG levels in ADH/WT and ADH/TO-DKH brains were examined immunohistochemically using an anti-8-oxo-dG antibody. To avoid detecting 8-oxoG in RNA, brain slices were pretreated with RNase, then reacted with the anti-8-oxo-dG antibody with or without hydrochloric acid (HCl) denaturation. In both conditions, 8-oxo-dG IR was stronger in ADH/TO-DKH brains compared with ADH/WT brains (Fig. 4a, b), and the IR in ADH/TO-DKH brain was greatly reduced by pretreatment with MutM, a bacterial 8-oxoG DNA glycosylase (Supplementary Fig. S4a), indicating that these 8-oxo-dG IRs represent 8-oxoG in DNA. Nuclear 8-oxo-dG IR was detected with HCl treatment (Supplementary Fig. S4b upper panels) and was mostly co-localized with DAPI-stained nuclear DNA (nDNA) (Supplementary Fig. S4c). In contrast, cytoplasmic 8-oxo-dG IR was evident without HCl treatment (Supplementary Fig. S4b lower panels), and was co-localized with TFAM, a mitochondrial DNA (mtDNA) binding protein (Supplementary Fig. S4d).

Quantitative measurement of 8-oxo-dG IR revealed significantly increased 8-oxoG accumulation in nDNA in ADH/TO-DKH brains, being > 1.7-fold higher in the cortex and 1.5-fold higher in the DG, compared with ADH/WT brains (Fig. 4a, b, Supplementary Fig. S5). On the other hand, there was no significant difference in 8-oxoG accumulation in nDNA between ADH/WT and NT brains (Supplementary Fig. S6a, b). ADH/TO-DKH brains had a 1.3-fold increase in mitochondrial 8-oxoG levels in the cortex, but not in the DG, compared with ADH/WT brains. These results indicate that MTH1/OGG1 deficiency causes a significant increase in 8-oxoG accumulation in nDNA, and to a lesser extent in mtDNA, in ADH/TO-DKH brains.

These results indicate that ADH/TO-DKH brains undergo neurodegeneration accompanied by significantly increased accumulation of intracellular Aβ and 8-oxoG, especially in nDNA, indicating that a build-up of 8-oxoG in nDNA in the cortex and/or hippocampus accelerates AD pathology. Thus, *Mth1*/*Ogg1* knockout in 3xTg-AD-H mice reproduces the neurodegenerative process, a main pathological feature of the AD.

We reported that damaged neurons accumulate higher levels of 8-oxoG in mtDNA than in nDNA under oxidative conditions in the brain, while activated microglia mainly accumulate 8-oxoG in nDNA^27^. The former causes mitochondrial dysfunction and calpain-dependent neuronal dysfunction, and the latter results in poly (ADP-ribose) polymerase (PARP)-dependent activation of apoptosis-inducing factor (AIF) and exacerbated microgliosis (Fig. 4c)^3,27^.

To clarify whether mtDNA or nDNA accumulation of 8-oxoG is associated with neurodegeneration in the ADH/TO-DKH brain, we examined the extent of mitochondrial dysfunction by cytochrome c oxidase (COX) activity staining (Supplementary Fig. S7). Compared with ADH/WT brains, relative COX activity was significantly increased rather than decreased in cortex ADH/TO-DKH and was not changed in hippocampus, indicating that MTH1/OGG1 deficiency did not exacerbate mitochondrial dysfunction. Electron microscopy analysis (Supplementary Fig. S8) revealed that ADH/TO-DKH mice did not exhibit reduced mitochondrial numbers in the cortex or hippocampus compared with NT mice. Moreover, mitochondria in the ADH/TO-DKH cortex showed intact matrices, supporting the COX activity staining results. In contrast, we found a significant increase in IR for CD68, a marker for activated microglia, in the cortex and to a lesser extent in the DG of ADH/TO-DKH compared with ADH/WT mice (Fig. 4d). Microglia in the ADH/TO-DKH brain were therefore activated; however, microglial density was unchanged (Supplementary Fig. S9a). As shown in Fig. 4e, strong nuclear 8-oxo-dG IR was also detected in Iba1-positive microglia in the ADH/TO-DKH cortex.

These results indicate that MTH1/OGG1 deficiency in the ADH/TO-DKH brain increases accumulation of 8-oxoG in microglial nDNA and causes microglial activation.

### Minocycline, an inhibitor of microglial activation, markedly decreases the nuclear accumulation of 8-oxoG and PARP activation in microglia, and neurodegeneration

**Figure 5:**
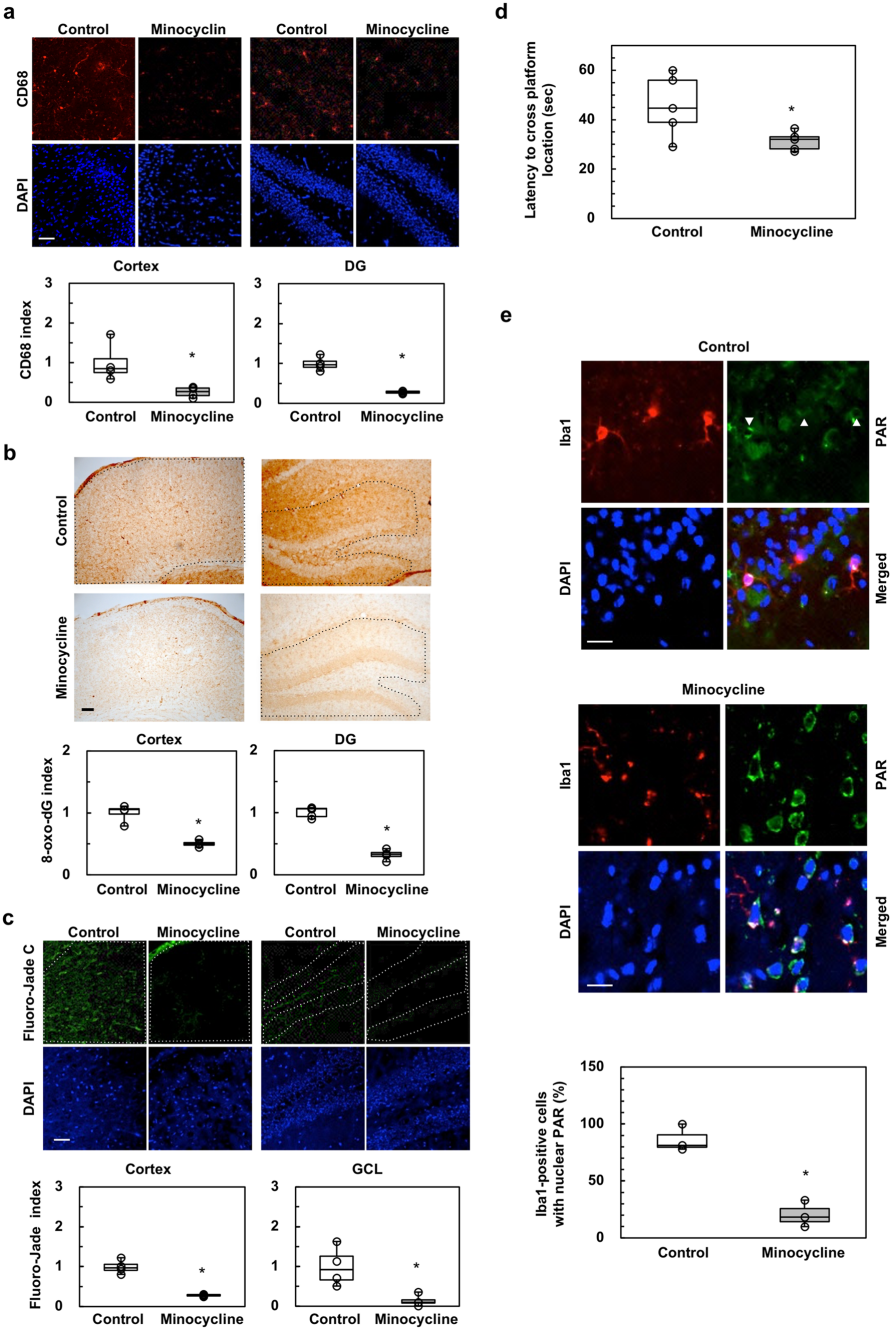
Microglial activation caused by nuclear 8-oxoG accumulation is responsible for neurodegeneration in ADH/TO-DKH brains. (**a**) Minocycline reduced the CD68 index in ADH/TO-DKH brains. Scale bar = 50 µm. Control, no treatment. *n* = 4. Wilcoxon exact test (two-sided), *p = 0.0286. (**b)** Minocycline reduced nuclear 8-oxo-dG index in ADH/TO-DKH brains. Scale bar = 100 µm. *n* = 4. Wilcoxon exact test (two-sided), *p = 0.0286. (**c**) Minocycline reduced Fluoro-Jade C index in ADH/TO-DKH brains. Scale bar = 50 µm. n = 4. Wilcoxon exact test (two-sided), *p = 0.0286. (**d**) MWM test (probe test) after minocycline administration to ADH/TO-DKH. Latencies to cross the platform location 24 h after the training trial are shown. *n* = 5. Wilcoxon exact test (one-sided), *p = 0.0286. (**e**) Minocycline reduced nuclear PAR positive microglia in ADH/TO-DKH cortex. Arrowheads indicate Iba1-positive cells with nuclear PAR. Scale bar = 20 µm. Number of Iba1-positive cells with nuclear PAR in the cortex was counted. *n* = 3. Wilcoxon exact test (one-sided), *p = 0.05.

To examine whether microglial activation is responsible for the neurodegeneration in ADH/TO-DKH brains, we administered minocycline, which inhibits microglia activation and cytokine responses^32^, to ADH/TO-DKH mice for 3 weeks. As shown in Fig. 5a, much less CD68 IR was detected in both the cortex and the DG of ADH/TO-DKH mice after minocycline treatment compared with control mice. Microglial density was unchanged by the treatment (Supplementary Fig. S9b). Increased nuclear 8-oxo-dG IR in both the cortex and DG of ADH/TO-DKH mice was also markedly decreased after minocycline treatment (Fig. 5b).

Minocycline treatment significantly decreased green fluorescence derived from bound Fluoro-Jade C in the cortex and GCL of the DG in ADH/TO-DKH mice (Fig. 5c), indicating that suppression of microgliosis ameliorates neurodegeneration caused by *Mth1*/*Ogg1* knockout. MWM test (probe test) revealed a significant decrease in the initial latency to cross the platform location, indicating that cognitive dysfunction was also improved (Fig. 5d). In ADH/TO-DKH brains, immunofluorescent signals for poly (ADP-ribose) (PAR) were highly accumulated in the nuclei of Iba1-positive microglia in the absence of minocycline treatment (Fig. 5e, upper panel and graph). Minocycline treatment significantly decreased the number of Iba1-positive microglia with nuclear PAR in the ADH/TO-DKH cortex (Fig. 5e, lower panel and graph).

These results indicate that microglial activation in the ADH/TO-DKH brain, which is associated with 8-oxoG accumulation in microglial nDNA and PARP activation, is responsible for the neurodegeneration and cognitive dysfunction.

### Expression of neuroprotective genes is altered in the hippocampus of ADH/TO-DKH mice

**Figure 6:**
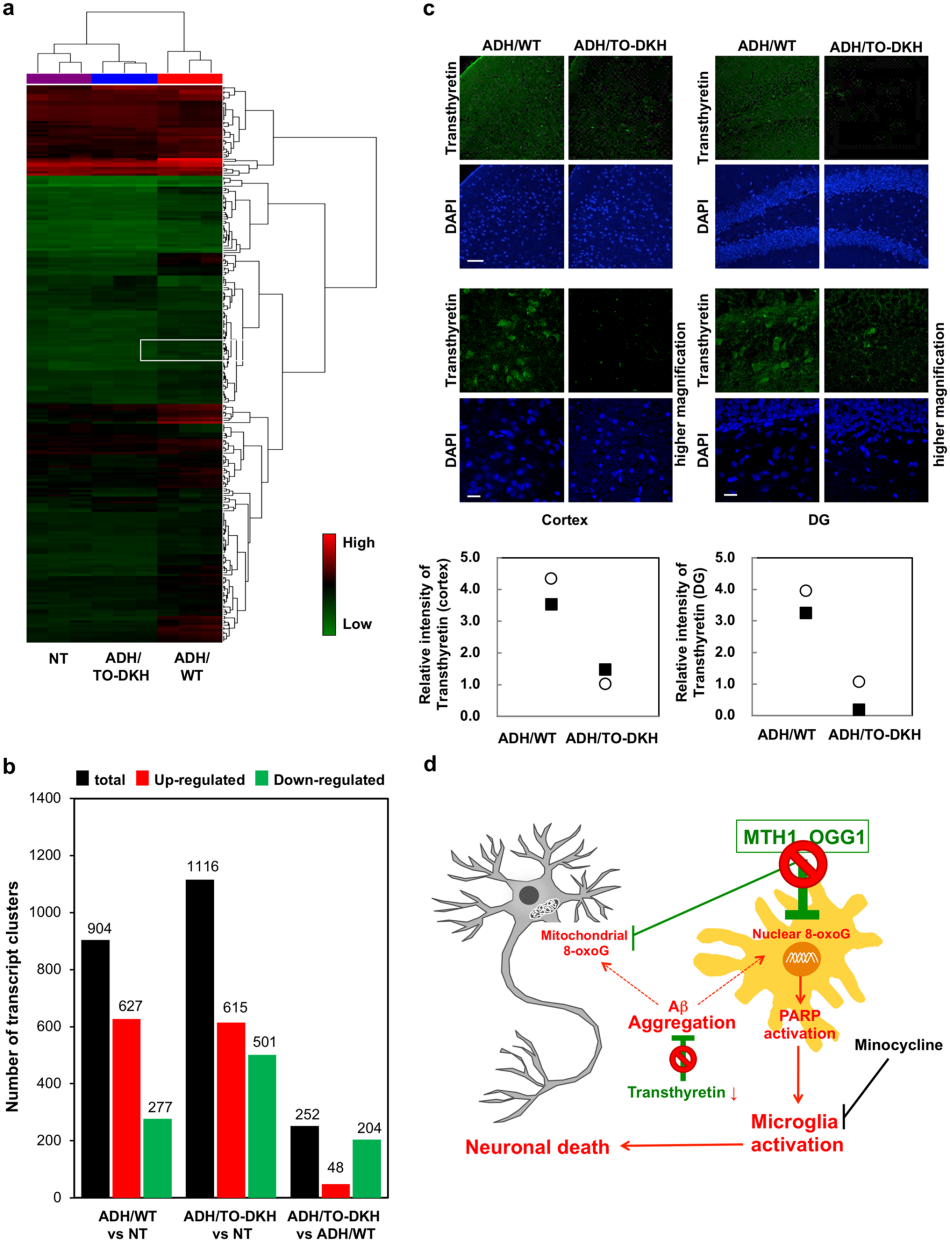
Altered gene expression profiles in the hippocampus of ADH/TO-DKH mice. (**a**) Hierarchical and partitioning clustering identified 1687 transcript clusters with a > 1.5-fold difference in expression (eBayes ANOVA, *p* < 0.05, *n* = 3) among NT, ADH/WT and ADH/TO-DKH hippocampi. In the heat map, green to red represents low to high expression. (**b**) Numbers of transcript clusters whose expression levels were significantly altered between ADH/WT and NT, ADH/TO-DKH and NT, and ADH/TO-DKH and ADH/WT, respectively. (**c**) Immunodetection of transthyretin in cortex (left panels) and DG (right panels). Nuclei were stained with DAPI. Scale bar = 50 µm. Higher magnification images are shown in the lower panels. Scale bar = 20 µm. The signal intensities of transthyretin in two individual mice, different from those used for the gene expression profiles, were analyzed for each of ADH/WT and ADH/TO-DKH, and are shown as dot graphs. (**d**) MTH1/OGG1 play a pivotal role in suppression of nuclear 8-oxoG accumulation in microglia. *Mth1*/*Ogg1* knockout causes a vicious cycle of microgliosis resulting in severe neurodegeneration, which in turn also increases intracellular Aβ accumulation by transthyretin downregulation. Accumulation of Aβ produces ROS and accelerates the cycle.

To delineate the mechanism underling the neurodegeneration, we performed gene expression profiling using microarray analysis of RNA prepared from NT, ADH/WT and ADH/TO-DKH hippocampi. As a result, 1687 transcript clusters showed a difference among the three groups (Fig. 6a, b, Supplementary Table 1). However, hierarchical clustering revealed that the expression of very few gene clusters are altered between ADH/TO-DKH and ADH/WT, suggesting the limited genes may play a major role in neurodegeneration (shown as a white line in Fig. 6a). The 38 genes including neuroprotective genes, such as *Ttr*, *Enpp2*, *Kcnj13* and *Kl* (Table 1), whose expression is significantly upregulated as part of the protective response to increased levels of mutant Tau and/or APP proteins, were significantly downregulated in the ADH/TO-DKH^33,34^. These results suggest that downregulation of neuroprotective genes is responsible for the early onset of neurodegeneration. The expression level of *Ttr*, which encodes transthyretin, is most significantly altered among the three groups. Transthyretin is a major Aβ-binding protein that acts as a neuroprotector in AD by suppressing aggregation of Aβ^35^ or proteolytically cleaving Aβ^36^. Therefore, we compared expression levels of transthyretin between ADH/TO-DKH and ADH/WT brains. Immunofluorescence microscopy using an anti-transthyretin antibody revealed significantly reduced levels of transthyretin in the cortex and DG of ADH/TO-DKH brains compared with ADH/WT brains (Fig. 6c, upper panel and graph).

**Table 1:** List of genes with significantly different expression between ADH/TO-DKH and ADH/WT hippocampi.

Gene Symbol^#^	Expression level (raw intensity)			ADH/TO-DKH vs ADH/WT			ADH/WT vs NT		ADH/TO-DKH vs NT		REF*
	NT	ADH/WT	ADH/ TO-DKH	Expression change	Fold Change	*p*-val	Fold Change	*p*-val	Fold Change	*p*-val	
*Ttr*	984.41	14,235.25	806.34	− 13,428.91	− 17.65	8.39E-07	14.46	1.47E-06	− 1.22	4.48E-01	34, 35
*Enpp2*	1616.00	7703.99	1786.64	− 5917.35	− 4.31	3.10E-07	4.77	2.06E-07	1.11	5.13E-01	34, 35
*Kcnj13*	146.36	2745.91	76.53	− 2669.38	− 35.88	9.66E-08	18.76	4.83E-07	− 1.91	2.40E-02	35
*Kl*	201.89	1086.50	230.90	− 855.60	− 4.71	8.52E-08	5.38	3.59E-08	1.14	1.25E-01	33, 34, 35
*Ace*	93.62	630.45	125.42	− 505.03	− 5.03	6.74E-05	6.73	1.78E-05	1.34	2.20E-01	
*Sostdc1*	105.15	607.70	116.59	− 491.10	− 5.21	2.74E-07	5.78	2.42E-07	1.11	8.41E-01	33, 35
*Cd59a*	160.67	866.81	387.18	− 479.63	− 2.24	1.60E-03	5.39	3.99E-06	2.41	3.79E-04	
*Trpm3*	303.02	857.32	379.58	− 477.74	− 2.26	1.00E-04	2.83	1.71E-05	1.25	9.45E-02	33, 35
*Gpx8*	132.45	634.37	158.97	− 475.40	− 3.99	7.69E-07	4.79	2.12E-07	1.20	7.31E-02	
*Folr1*	93.38	541.20	73.07	− 468.13	− 7.41	4.84E-09	5.80	1.30E-08	− 1.28	3.98E-02	33, 34, 35
*Slc2a12*	116.72	488.63	153.90	− 334.73	− 3.17	5.05E-07	4.19	1.18E-07	1.32	3.86E-02	
*Clic6*	34.88	360.08	29.33	− 330.75	− 12.28	4.89E-08	10.32	8.24E-08	− 1.19	3.41E-01	33, 35
*Lbp*	120.68	432.24	105.19	− 327.05	− 4.11	3.22E-06	3.58	4.16E-06	− 1.15	7.58E-01	33, 34, 35
*Otx2*	49.47	341.26	36.31	− 304.96	− 9.40	1.07E-07	6.90	2.55E-07	− 1.36	1.64E-01	33, 35
*Prlr*	31.20	301.87	19.25	− 282.62	− 15.68	3.20E-07	9.67	9.55E-07	− 1.62	1.30E-01	33, 34, 35
*Slc4a5*	49.72	327.83	47.24	− 280.59	− 6.94	6.05E-07	6.59	1.60E-06	− 1.05	1.97E-01	34
*Steap2*	128.27	428.44	162.31	− 266.13	− 2.64	3.81E-05	3.34	7.02E-06	1.27	1.05E-01	
*Cab39l*	217.13	508.34	264.85	− 243.49	− 1.92	2.65E-05	2.34	3.21E-06	1.22	4.08E-02	
*Rdh5*	94.97	350.28	109.86	− 240.42	− 3.19	2.95E-07	3.69	1.13E-07	1.16	1.35E-01	33, 35
*Tmem72*	60.48	278.73	46.79	− 231.94	− 5.96	1.42E-06	4.61	4.80E-06	− 1.29	1.50E-01	
*Cldn1*	80.30	315.74	87.71	− 228.03	− 3.60	5.54E-06	3.93	2.61E-06	1.09	3.74E-01	33
*Slc4a2*	132.24	385.55	175.84	− 209.71	− 2.19	1.00E-04	2.92	9.32E-06	1.33	3.51E-02	33
*Igfbp2*	122.21	341.36	137.64	− 203.72	− 2.48	3.39E-05	2.79	1.04E-05	1.13	2.47E-01	33, 34
*Sulf1*	79.79	268.78	76.92	− 191.86	− 3.49	8.28E-07	3.37	1.07E-06	− 1.04	7.18E-01	33, 35
*Dab2*	240.88	318.36	136.09	− 182.27	− 2.34	4.24E-05	1.32	1.34E-02	− 1.77	1.81E-03	
*Calml4*	60.80	236.41	56.19	− 180.22	− 4.21	2.28E-05	3.89	2.42E-05	− 1.08	9.53E-01	33, 35
*Col8a1*	93.40	236.34	60.43	− 175.91	− 3.91	2.56E-06	2.53	1.77E-05	− 1.55	5.00E-02	33, 34, 35
*C1qtnf5*	102.35	260.42	87.77	− 172.65	− 2.97	9.30E-08	2.54	3.19E-07	− 1.17	6.18E-02	
*F5*	32.26	203.26	32.91	− 170.35	− 6.18	5.44E-07	6.30	5.84E-07	1.02	9.16E-01	35
*Pon3*	64.52	224.37	67.54	− 156.83	− 3.32	3.19E-06	3.48	1.42E-06	1.05	3.12E-01	
*Pcolce2*	130.51	317.52	165.02	− 152.50	− 1.92	2.00E-04	2.43	3.98E-05	1.26	1.61E-01	
*Pla2g5*	68.85	208.07	66.84	− 141.24	− 3.11	7.72E-06	3.02	1.39E-05	− 1.03	5.29E-01	
*Abca4*	50.42	176.06	45.88	− 130.18	− 3.84	2.48E-06	3.49	5.79E-06	− 1.10	3.20E-01	
*Drc7*	60.14	191.55	63.95	− 127.61	− 3.00	8.94E-07	3.18	7.69E-07	1.06	8.30E-01	
*Car14*	114.18	219.86	96.04	− 123.82	− 2.29	7.09E-05	1.93	1.67E-04	− 1.19	4.66E-01	
*Tc2n*	21.09	141.21	20.55	− 120.66	− 6.87	1.90E-05	6.70	2.08E-05	− 1.03	9.28E-01	
*Aqp1*	77.70	176.48	61.09	− 115.39	− 2.89	2.34E-06	2.27	1.29E-05	− 1.27	7.22E-02	33, 35
*A2m*	77.40	244.90	142.93	− 101.97	− 1.71	2.42E-02	3.16	3.14E-05	1.85	7.26E-04	
*Spink8*	53.90	129.47	278.78	149.31	2.15	6.20E-03	2.40	4.15E-03	5.17	4.56E-05	

Genes with significantly different expression between ADH/WT and ADH/TO-DKH hippocampi are shown in order of expression change between the two groups, with comparison to data from NT (eBayes ANOVA: false discovery rate [FDR] *p* < 0.05, fold-change > 1.5 or < − 1.5, highest raw intensity among three groups > 100).

*Altered expression of the corresponding gene was reported in hippocampus prepared from different mouse lines carrying neuron-specific enolase/human *Tau23* transgene ^33^, *APP*_swe_ transgene ^34^ or neuronal conditional deletion of *App* and *Aplp*2 ^35^.

## Discussion

In the present study, we established a novel mouse model of AD with neurodegeneration at 4 to 5 months of age. During aging, 3xTg-AD mice with *Mth1*/*Ogg1* wild-type alleles exhibit neuronal mitochondrial dysfunction^10^, probably because of increased 8-oxoG accumulation in mtDNA, but not neuronal loss or neurodegeneration^30,37,38^. Moreover, we reported that cortical neurons isolated from adult MTH1/OGG1-deficient mice accumulate high levels of 8-oxoG in mtDNA but not nDNA under in vitro oxidative conditions, and thus exhibit mitochondrial dysfunction and impaired neuritogenesis but not neuronal death^39^. These findings indicate that the mitochondrial dysfunction in neurons is not sufficient to cause neurodegeneration. Thus, MTH1 and OGG1 together maintain a low level of 8-oxoG during progression of AD, especially in microglial nDNA, thereby avoiding harmful activation of microglia and neurodegeneration (Fig. 6d).

Damaged neurons release find-me or eat-me signals which in turn activate surrounding microglia^15,40^. In the MTH1/OGG1-deficient AD mouse brain, significantly increased accumulation of 8-oxoG in nDNA of activated microglia was found in both the cortex and hippocampal DG, where severe neurodegeneration occurred (Fig. 1, 4). Activation and proliferation of microglia is dependent on ROS produced by microglial NADPH oxidase^41,42^; therefore, 8-oxo-dGTP formation in the nucleotide pool or oxidation of guanine in DNA must be enhanced in activated microglia, resulting in increased 8-oxoG accumulation, especially in replicating nDNA of activated microglia in the absence of MTH1 and OGG1 (Fig. 4c). Activated microglia can cause neuronal loss through phagocytosis of damaged neurons or even healthy neurons together with a neuroinflammatory response^40^. During phagocytosis, microglia produce further ROS, thereby enhancing 8-oxoG accumulation in nDNA of activated microglia, resulting in chronic activation of microglia, namely microgliosis^43^. It is likely that *Mth1*/*Ogg1* knockout ultimately causes a vicious cycle of microgliosis resulting in severe neurodegeneration^15^, which in turn also increases intracellular Aβ accumulation. Aβ itself causes mitochondrial dysfunction and increases ROS production during development of AD pathology (Fig. 6d).

As shown in Fig. 4a and b, 8-oxoG accumulation in mtDNA was only slightly higher in the cortex, but not the hippocampus, of ADH/TO-DKH mice compared with ADH/WT mice. This finding suggests that MTH1/OGG1 deficiency might not impair mitochondrial function in mice with a 3xTg-AD background. Our COX activity staining (Supplementary Fig. S7) and electron microscopy (Supplementary Fig. S8) results also support this observation. Furthermore, there was rather increased COX staining in the cortex of ADH/TO-DKH mice, suggesting that a small increase in mtDNA 8-oxoG may induce some protective responses; however, further investigation is needed to confirm this idea.

Limitations of our study are sample size and selection bias for analyses, because ADH/TO-DKH mice with longer latency times (> 40 s) and ADH/WT mice with shorter latency times (< 20 s, similar to NT mice) on day 5 of the MWM were subjected to pathological examination in order to examine histopathological changes in mice with cognitive impairment. We observed that levels of 8-oxoG accumulation in nuclear DNA, which are expected to be suppressed by MTH1/OGG1, are consistently parallel to Fluoro-Jade C index, densities of hematoxylin-stained nuclei and TUNEL-positive cells as well as Aβ index in both cortex and GCL, even with minocycline treatment. However, it remains to clarify whether all ADH/TO-DKH mice exhibit essentially the same results, because some showed shorter latency (< 40 s) in MWM test.

In conclusion, the present study provides experimental evidence for efficient suppression of 8-oxoG accumulation in AD brains by MTH1 and OGG1. However, their deficiency causes microglial activation resulting in neurodegeneration, which is known to occur in the late stage of sporadic AD, and suggests possible new approaches for prevention and treatment of AD.

## Methods

### Animals

Homozygous triple transgenic AD model mice (3xTg-AD-H) carrying a homozygous *Psen1*_M146V_ knock-in mutation, and homozygous mutant transgenes for *APP*_Swe_ and *MAPT*_P301L_ were previously established^29^. 3xTg-AD-H mice were backcrossed once to C57BL/6 J mice (Clea Japan, Tokyo, Japan). The hemizygous 3xTg-AD (3xTg-AD-h) mice obtained were used to re-establish 3xTg-AD-H mice^44^. We previously established *Mth1* and *Ogg1* gene knockout mice^45,46^ and heterozygotes (*Mth1*^+/−^ and *Ogg1*^+/−^) have been backcrossed to C57BL/6 J for more than 18 generations. 3xTg-AD-h mice carrying heterozygous mutant alleles (*Mth1*^+/–^/*Ogg1*^+/–^) were obtained by mating 3xTg-AD-H and *Mth1*^–/–^/*Ogg1*^–/–^ mice. These were further inbred, and the following mouse lines were established: NT, carrying neither a transgene nor mutant allele; ADH/WT, 3xTg-AD-H mice carrying homozygous wild-type alleles (*Mth1*^+/+^ and *Ogg1*^+/+^); and ADH/TO-DKH, 3xTg-AD-H mice carrying homozygous mutant alleles (*Mth1*^–/–^ and *Ogg1*^–/–^). It has been reported that Alzheimer’s disease-related cognitive function is modified by the ovarian cycle^47^. Thus, to clarify the effects of MTH1/OGG1 deficiency on cognitive function, we used only male mice at 4 to 5 months of age, NT (22 week-old), ADH/WT (18 to 20-week old), and ADH/TO-DKH (16 to 18-week old) for the experiments. All animals were maintained in an air-conditioned, specific pathogen-free room with time-controlled lighting. All animal experimental procedures were reviewed and approved by Animal Care and Use Committee at Kyushu University (approval numbers A20-080–0, A-30–077-0, A29-116–0, A28-013–0, A27-257–0, A26-011–0), and were carried out in accordance with relevant guidelines and regulations. All animal experimental protocols were performed with adherence to ARRIVE (Animal Research: Reporting of In Vivo Experiments) guidelines (https://www.nc3rs.org.uk/arrive-guidelines).

### Genotyping

Alkaline extracted DNA from tail tissue biopsies was used for genomic PCR using MightyAmp DNA Polymerase Ver. 2 (Takara Bio, Shiga, Japan), according to the manufacturer’s instructions. For genotyping the 3xTg-AD mice, the *Psen1*_M146V_ knock-in mutation was detected as previously described^48^. The *APP*_Swe_ and *MAPT*_P301L_ transgenes were identified using specific primer sets: *APP*_Swe_, 5′-GAGGTATTCAGTCATGTGCT-3′, 5′-GCTTGCACCAGTTCTGGATGG-3′; *MAPT*_P301L_, 5′-GAGGTATTCAGTCATGTGCT-3′, 5′-TTCAAAGTTCACCTGATAGT-3′. To genotype the *Mth1* and *Ogg1* alleles, specific primer sets were used: *Mth1*^+^ allele (749 bp): 5′-CTCTCCAGCCCTTGTTCAAGTTC-3′ and 5′-CCTACTCTCTTGGGCTTCATCC-3′; *Mth1*^–^ allele (814 bp): 5′-CTCTCCAGCCCTTGTTCAAGTTC-3′ and 5′-GAACCTGCGTGCAATCCATCTTGT-3′; Ogg1 + allele (829 bp): 5′-GTTAAGCTTCAAACGTGCCTC-3′ and 5′-GAAGGACTGTCCAGAAGCTA-3′; *Ogg1*^–^ allele (644 bp): 5′-GTTAAGCTTCAAACGTGCCTC-3′ and 5′-CTACGCATCGGTAATGAAGG-3′^39^. Homozygosity of the *APP*_Swe_ transgenes was determined by quantitative real time PCR using the Thermal Cycler Dice Real Time System Single (Takara) and purified tail DNA with the primer sets: *APP*_Swe_: 5′-ATTCAGATCCATCAGGGACCAA-3′ and 5′-GCTTGCACCAGTTCTGGATGGT-3′. As a control, a primer set for RNase P was used: 5′-GCCGGAGCTTGGAACAGA-3′ and 5′-GGTGCCTCACCTCAGCCAT-3′. Each reaction was performed with 25 ng of tail DNA, 200 nM primers and 12.5 μl 2 × SYBR green ready reaction mix with Rox (Applied Biosystems, Foster City, CA, USA) in a total volume of 25 μl.

### Behavioral analysis—Morris water maze

The water maze consisted of a circular polyvinyl chloride pool (100 cm in diameter and 30 cm high) that was filled to a depth of 15 cm with room-temperature tap water (25 ± 1 °C). The performance of the mice was scored using a video camera-based computer tracking system, Watermaze 3 (Actimetrics, Wilmette, IL, USA) on a Sony computer, with the camera fixed to the ceiling 2 m above the pool. To evaluate spatial working memory, mice were given three training trials a day for 5 days (non-cued test). Mice were trained to escape onto a 15 cm diameter, clear, circular Plexiglas platform submerged 1 cm beneath the surface of the water that was invisible to the mice while swimming. The platform was kept in the same location throughout training^10^. On each trial, the mouse was placed into the pool at one of four designated start points. Mice were allowed to find and escape onto the submerged platform. If a mouse failed to find the platform within 60 s, it was manually guided to the platform and allowed to remain there for 15 s. After this, each mouse was placed into a holding cage under a warming lamp for 60 s until the start of the next trial^49^. Initial latency to escape onto the platform and total swimming distance were measured. To evaluate memory retrieval, a probe test was conducted in the pool without the platform for 60 s, 1.5 and 24 h after the three acquisition trials. The parameters measured during the probe test were as follows: (1) initial latency to cross the platform location, (2) time spent in target quadrant during the test period, and (3) number of crossings of the platform location^50^.

### Tissue processing

To examine histopathological changes in mice with cognitive impairment, ADH/TO-DKH mice with longer latency times (> 40 s) and ADH/WT mice with shorter latency times (< 20 s, similar to NT mice) on day 5 of the MWM were subjected to pathological examination. Mice were deeply anesthetized with pentobarbital (75 mg/kg i.p.), and perfused intracardially with saline followed by cold 4% paraformaldehyde (PFA) in phosphate-buffered saline (PBS). Brains were removed, immersed in 4% PFA for 12 h followed by 20% and 30% sucrose in PBS for 24 h each at 4 °C. Brains were then embedded in Tissue-Tek O.C.T. compound (Sakura Finetek, Tokyo, Japan), frozen and stored at − 80 °C, or embedded in paraffin and stored at 4 °C.

### Antibodies

Mouse monoclonal anti-human Aβ N-terminal-specific antibody (clone 82E1), which recognizes the N-terminus of the processed form, but not the APP protein, was obtained from Immuno-Biological Laboratories (Gunma, Japan). Rabbit polyclonal anti-Iba1 (019–19,741), a microglia marker, was obtained from Wako Pure Chemical Industries (Osaka, Japan). Rat monoclonal anti-CD68 (MCA1957), a marker for activated microglia, was obtained from BIO-RAD Laboratories (Berkeley, CA, USA). Rabbit polyclonal anti-neuron-specific nuclear protein (NeuN; ABN78) was purchased from Millipore (Billerica, MA, USA). Mouse monoclonal anti-poly-ADP-ribose polymers (PAR; MC-100) were obtained from Trevigen (Gaithersburg, MD, USA). Mouse monoclonal anti-8-oxo-7,8-dihydro-2′-deoxyguanosine (anti-8-oxo-dG) (N45.1) was purchased from the Japan Institute for the Control of Aging, Nikken SEIL (Shizuoka, Japan). Mouse monoclonal anti-prealbumin (E-1, sc-377517), which recognizes transthyretin, was obtained from Santa Cruz Biotechnology (Dallas, TX, USA).

### Quantitative immunohistochemistry and immunofluorescence microscopy

To detect Aβ peptide immunohistochemically (Fig. 3a), paraffin-embedded blocks were cut on a microtome (4 μm thick) and mounted from warm water (42 °C) onto slides. After drying overnight at room temperature, sections were deparaffinized and citrate buffer antigen retrieval (10 mM sodium citrate, pH 6.0) was performed at 120 °C for 5 min. Sections were then blocked with a solution containing 1 × Block Ace for 30 min at room temperature, incubated with a primary antibody in 0.1 × Block Ace at 4 °C overnight. The DAB reaction was used to visualize the bound secondary antibody. Digital images were acquired using an Axioskop2 Plus microscope, equipped with an AxioCam CCD camera and Axiovision 3.1 imaging software (Carl Zeiss MicroImaging, Tokyo, Japan). An entire image of a coronal brain section was acquired using a Nikon eclipse 80i microscope (Nikon, Co., Tokyo, Japan) equipped with Stereo Investigator 10, Virtual Slice Module (MBF Bioscience Japan, Chiba, Japan). The intensity of immunoreactivity (IR) was measured in each digital image using ImageJ 1.49 V (National Institutes of Health, Bethesda, MD, USA). To detect Aβ peptide, CD68, NeuN, PAR and transthyretin, frozen tissue blocks were cut on a cryostat (40 μm thick), and collected as free-floating sections in PBS. Tissue sections were pre-incubated for 2 h with 10% normal goat serum in PBS followed by 0.1 × Block Ace for 2 h and then incubated with a primary antibody. These treated sections were incubated with the appropriate Alexa Fluor-labeled second antibody in a solution containing 0.05 μg/ml 4′,6-diamidino-2-phenylindole (DAPI) (Sigma-Aldrich Japan, Tokyo, Japan), which stained nuclear DNA, for 45 min at room temperature. Fluorescent images were acquired using an LSM700 Laser Scanning Microscope (Carl Zeiss MicroImaging). To obtain the relative index of immunofluorescence intensity for Aβ and CD68, each image was converted to binary monochrome using Adobe PhotoShop CS5 (Adobe Systems, San Jose, CA, USA), and then signal intensity was measured in a given area using ImageJ 1.49 V, and the intensity per unit area was defined as Aβ or CD68 index, and is shown relative to that in ADH/WT (Fig. 3b, Fig. 4d) or control (Fig. 5a).

### Western blotting for Aβ peptide

Mice were euthanized by cervical dislocation and their brains were removed and quickly frozen in liquid nitrogen. Frozen mouse cortices were lysed in 2 × sodium dodecyl sulfate (SDS) buffer containing 125 mM Tris–HCl (pH 6.8), 4% SDS, 4% 2-mercaptoethanol, 10% glycerol, 1% protease inhibitor cocktail, and 1% phosphatase inhibitor cocktail (25,955–11 and 07,574–61, respectively, Nacalai Tesque, Inc., Kyoto, Japan) followed by sonication. The homogenates were then centrifuged at 100,000 × *g* for 30 min at 20 °C using an Optima TLX ultracentrifuge and TLA55 rotor (Beckman Coulter Inc., Brea, CA, USA). The supernatant was stored at − 30 °C as the SDS-soluble fraction. The pellet was washed once with the same buffer and centrifuged at 100,000 × *g* for 10 min at 20 °C. Next, the pellet was dissolved in 100 µL of 70% formic acid (FA) by sonication. The FA-extracted samples were then centrifuged at 100,000 × *g* for 30 min at 20 °C. The supernatant was transferred to a new tube, and the FA was removed by freeze-drying (Savant SpeedVac, Thermo Fisher Scientific K.K., Tokyo, Japan). The pellet was then dissolved in dimethyl sulfoxide by sonication and stored at − 30 °C as the SDS-insoluble/FA-extractable fraction. For western blotting, samples were diluted with appropriate volumes of 2 × SDS buffer. Protein concentrations were determined using the XL-Bradford Protein Assay Reagent Kit (Integral Co., Ltd., Tokyo, Japan). Proteins were separated using a 12% Bis–Tris SDS-PAGE/MES SDS Running Buffer system (NP-0002, Thermo Fisher Scientific K.K.) on a Bio-Rad Mini PROTEAN 3 apparatus (Bio-Rad Laboratories K.K., Tokyo, Japan). Proteins separated on Bis–Tris gels were then transferred to nitrocellulose filters (Amersham Protran 0.2; 10,600,001, Cytiva, Tokyo, Japan) using 2 × NuPage Transfer Buffer (NP-0006, Thermo Fisher Scientific K.K.) and Trans-Blot Turbo (Bio-Rad Laboratories K.K.). After blocking with 5% skim milk in Tris-buffered saline + 0.1% Tween 20 for 1 h at room temperature, the filters were incubated with primary antibody overnight at 4 °C. To detect human Aβ peptides, we used mouse anti-human Aβ 82E1 antibody, which reacts with both soluble and fibrillar Aβ, but not with full-length APP. Horseradish peroxidase (HRP)-conjugated goat anti-mouse IgG (#7076, 1:5000, Cell Signaling Technology, Danvers, MA, USA), Takara Western Blot Quant HRP (T7102A), and EZ-Capture MG (ATTO, Tokyo, Japan) were used for chemiluminescence detection.

### Quantitative immunodetection of 8-oxo-7,8-dihydro-2´-deoxyguanosine (8-oxo-dG)

For the immunodetection of 8-oxo-dG in nuclear DNA (nDNA) or mitochondrial DNA (mtDNA), frozen tissue blocks were cut on a cryostat (40 μm thick) and collected as free-floating sections in PBS. The sections were pretreated as described previously ^51^. Briefly, to detect 8-oxo-dG in mtDNA, sections were pretreated with 5 mg/ml RNase (Sigma-Aldrich) only. To detect 8-oxo-dG in nDNA, sections were also pretreated with 2 N hydrochloric acid to denature the nDNA following RNase-treatment. Immunohistochemistry was then performed on the sections using the anti-8-oxo-dG antibody (1:100), the appropriate secondary antibody, and a Vector ABC kit, with visualization using DAB. Digital images were acquired using an Axioskop2 Plus microscope, equipped with an AxioCam CCD camera and Axiovision 3.1 imaging software. The intensity of the 8-oxo-dG IR was measured in each digital image using ImageJ 1.49 V. To obtain the relative 8-oxo-dG index, the intensity in the region enclosed by a dotted line was measured in each digital image and the intensity per unit area was defined and then shown as the relative index to that in ADH/WT (Fig. 4a, b) or control mice (Fig. 5b). An entire image of a coronal brain section was acquired as described above. To confirm the specificity of 8-oxo-dG IR, brain sections were pretreated with 5 mg/ml RNase, and then incubated with or without 10 μg/ml Escherichia coli MutM protein carrying 8-oxoG DNA glycosylase activity (F3174, Sigma-Aldrich) in nicking buffer [10 mM Tris–HCl (pH 7.5), 5 mM ZnCl2, 0.5 mM DTT, 0.5 mM EDTA, 1.5% glycerol, 100 μg/ml BSA] for 1 h at 37 °C. Sections were then subjected to immunohistochemistry or immunofluorescence microscopy with anti-8-oxo-dG and appropriate second antibodies.

### Histochemical detection of mitochondrial cytochrome c oxidase activity

To detect mitochondrial cytochrome c oxidase activity, frozen brain Sects. (100 μm thick) were incubated for 2.5 h at 37 °C in a solution containing cytochrome c (0.5 mg/ml), DAB (0.5 mg/ml), and sucrose (40 mg/ml) in PBS. The sections were then washed in PBS, dehydrated with ethanol, immersed in xylene and mounted on glass slides. Digital images were acquired using an Axioskop2 Plus microscope, equipped with an AxioCam CCD camera and Axiovision 3.1 imaging^10^.

### Detection of degenerating neurons

Fluoro-JadeC stains not only degenerating nerve cell bodies but also distal dendrites, axons and terminals. Fluoro-JadeC (AG325, Millipore) staining was performed according to the manufacturer’s instructions. To detect the DNA fragmentation that results from apoptotic signaling cascades, TUNEL (TdT-mediated dUTP nick end labeling) was performed using a TACS 2 TdT Kit HRP-DAB (4810-30-K, Trevigen) according to the manufacturer’s instructions. The number of TUNEL-positive nuclei in the area shown in Fig. 2 was counted.

### Minocycline treatment

ADH/TO-DKH mice were administered minocycline hydrochloride (M9511, Sigma-Aldrich) in the drinking water for 3 weeks to inhibit microglial activation. Minocycline was dissolved in the drinking water (0.5 mg/mL), and water bottles were changed daily. To confirm minocycline intake, the daily water intake was measured in four randomly selected mice. Reduced body weight was not observed in any of the treated mice. Compared with control (untreated) animals, water intake was unchanged in the treated mice. Daily minocycline intake was 0.13 ± 0.017 mg/g of body weight.

### Gene expression profiling with microarray analysis

Total RNA was isolated from the hippocampus using Isogen and 100 ng RNA was subjected to microarray analysis. Expression profiles were determined using Mouse Gene 1.0ST arrays (Affymetrix, Santa Clara, CA, USA) according to the manufacturer’s instructions^10^. To generate amplified and biotinylated sense-strand DNA targets from all expressed transcripts, the Ambion WT Expression Kit (Ambion, Austin, TX, USA) and the GeneChip WT Terminal Labeling and Controls Kit (Affymetrix) were used. CEL files generated were imported into Transcriptome Analysis Console (TAC) 4.0 Software (Affymetrix) and gene-level estimates were obtained. The gene-level estimates were further subjected to statistical analysis and hierarchical and partitioning clustering. All microarray data were deposited in the GEO database (accession number GSE83996).

### Statistical analysis

Statistical analysis of microarray data including hierarchical and partitioning clustering was performed using TAC4.0. Gene-level estimates of microarray data were subjected to one-way eBayes ANOVA, and the resulting list of transcript clusters with *p* < 0.05 with specific comparisons (e.g., ADH/TO-DKH vs ADH/WT, ADH/TO-DKH vs NT) with a fold-change > 1.5 or < − 1.5 as a threshold for comparison, was subjected to the hierarchical and partitioning clustering. All other experiments were analyzed using JMP Pro 14.3 and 15.2.1 software (SAS Institute Japan, Tokyo, Japan). The Wilcoxon exact test was used as a non-parametric method. Box plots are shown with a five-number summary: the minimum, the maximum, the sample median, and the first and third quartiles, in which the median is included.

## Supplementary Information

Supplementary Information 1.

Supplementary Information 2.

## Data Availability

All data except microarray data are available in the manuscript or the supplementary materials. All microarray data are deposited in the GEO database (accession number GSE83996).

## Code availability

Watermaze 3 (Actimetrics), Axiovision 3.1 imaging software (Carl Zeiss MicroImaging), Transcriptome Analysis Console (TAC) 4.0 Software (Affymetrix), ImageJ 1.49 V (National Institutes of Health), JMP Pro 14.3 (SAS Institute), and Adobe PhotoShop CS5 (Adobe Systems) were used for collection and analysis of data.
