# Supplementary material for: MTH1 and OGG1 maintain a low level of 8-oxoguanine in Alzheimer's brain, and prevent the progression of Alzheimer's pathogenesis
Source: Sci Rep. 2021 Mar 23;11:5819. doi: 10.1038/s41598-021-84640-9 (PMC7988129; doi:10.1038/s41598-021-84640-9)
Supplement: Supplementary file 1 — Supplementary Information 1. [file 41598_2021_84640_MOESM1_ESM.pdf]

Supplementary information

MTH1 and OGG1 maintain a low level of 8-oxoguanine in Alzheimer's brain, and prevent the progression of Alzheimer's pathogenesis

Sugako Oka[1†], Julio Leon[1‡], Kunihiko Sakumi[1], Nona Abolhassani[1], Zijing Sheng[1], Daisuke Tsuchimoto[1], Frank M. LaFerla[2], Yusaku Nakabeppu[1*]

[1]Division of Neurofunctional Genomics, Department of Immunobiology and Neuroscience, Medical Institute of Bioregulation, Kyushu University, Fukuoka, 812-8582, Japan
[2]Department of Neurobiology and Behavior, University of California, Irvine, CA 92697, USA

[†]Present address: Department of Cancer Biology, University of Kansas Medical Center, Kansas City, KS 66010, USA
[‡]Present address: Laboratory for Advanced Genomics Circuit, RIKEN Center for Integrative Medical Sciences, Yokohama, 230-0045, Japan

This file contains Supplementary Methods and Supplementary Figures S1 to S11.

Supplementary Methods

Transmission electron microscopy

The fixed male mouse brain, stored in O.C.T compound, was quickly thawed in 30% sucrose in PBS at 37°C. Small pieces of tissue were dissected from the cortex and hippocampus, and were then equilibrated to 3.4% sucrose in PBS using a six-step equilibration protocol (1 h each). Next, the tissue was fixed in glutaraldehyde (GA) solution (2.5% glutaraldehyde and 3.4% sucrose in 0.1 M phosphate buffer [pH 7.4]) overnight at 4°C. For female mice, the cortex of the frozen brain that was also used for western blotting was dissected and directly fixed in GA solution. After rinsing in PBS, all tissue was post-fixed with 1% osmium tetroxide for 2 h, dehydrated in ethanol and propylene oxide, and embedded in Epon resin (Epon 812 Resin Kit; TAAB Laboratories, Aldermaston, England). Ultrathin sections (80 nm) were stained with uranyl acetate for 5 min and lead acetate for 10 min, and were then examined using a transmission electron microscope (Tecnai 20; FEI Company Japan Ltd, Tokyo, Japan).

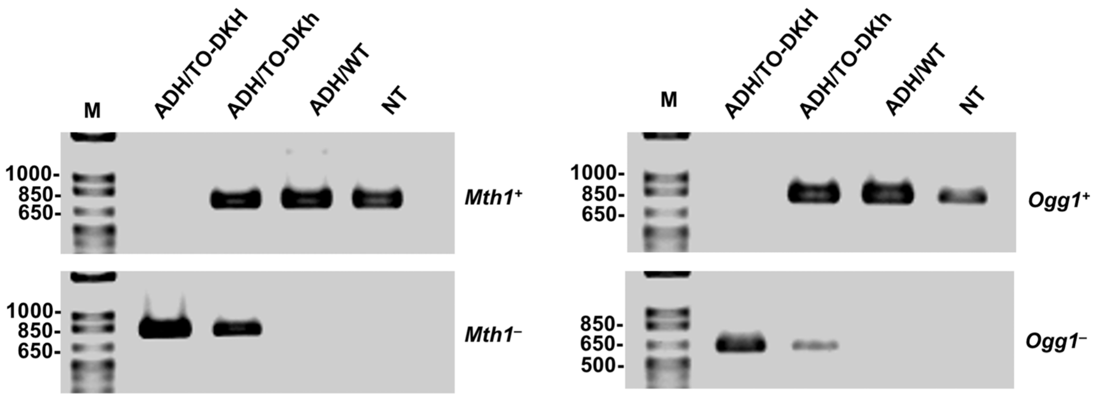

Supplementary Figure S1. Establishment and characterization of *Mth1*/*Ogg1*-double knockout AD model mice. Genotyping of *Mth1* and *Ogg1* alleles by genomic PCR. Full-length gels are presented in Supplementary Figure S11.

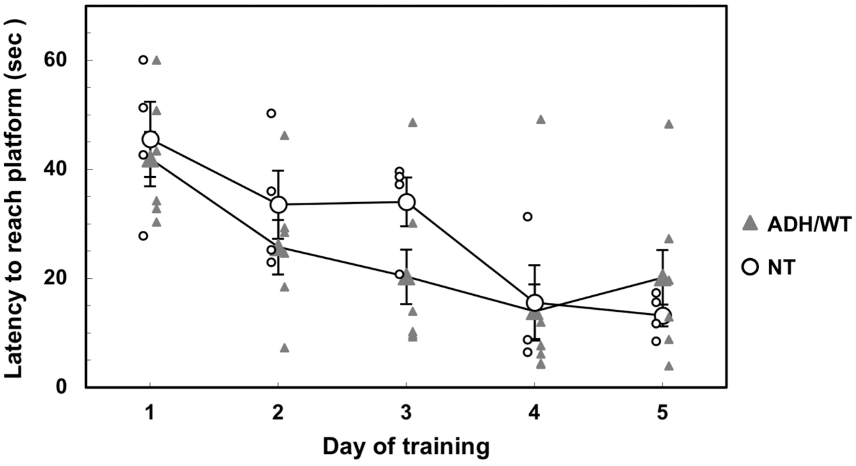

Supplementary Figure S2. Morris water maze test. MWM test (non-cued test). Latencies to reach the platform are shown. n = 4 – 6. Data are presented as the means ± SEM.

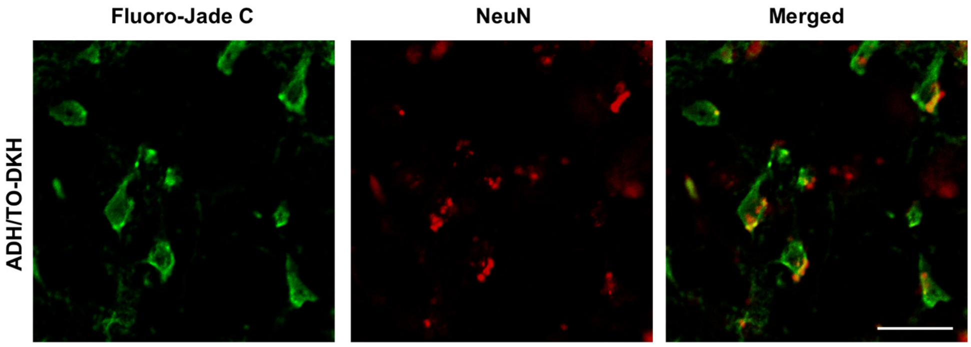

Supplementary Figure S3. *Mth1*/*Ogg1* knockout triggered neurodegeneration. Fluoro-Jade C mostly co-localized with the neuronal marker, NeuN, in the ADH/TO-DKH cortex. Scale bar = 20 μm.

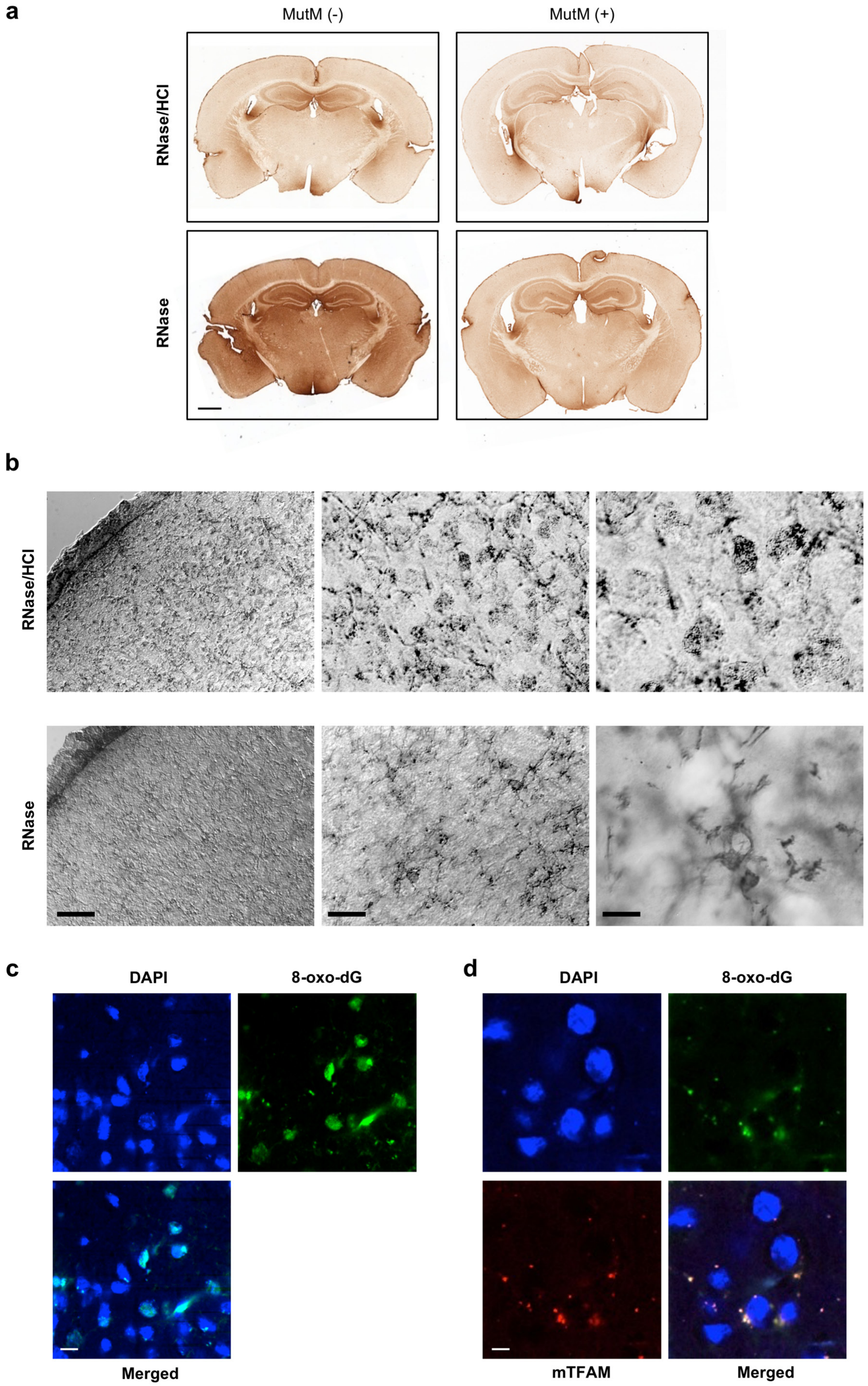
a
MutM (-)
MutM (+)
RNase/HCl
RNase
b
RNase/HCl
RNase
c
DAPI
8-oxo-dG
Merged
d
DAPI
8-oxo-dG
mTFAM
Merged

Supplementary Figure S4. Validation of the specificity of 8-oxo-dG immunoreactivity**.** (**a**) Pretreatment with MutM eliminates 8-oxog-dG immunoreactivity. Brain sections of 5-month-old ADH/TO-DKH mice were pretreated with 5 mg/ml RNase and then incubated without (–MutM) or with (+MutM) 10 μg/ml MutM protein (F3174, Sigma-Aldrich) in nicking buffer [10 mM Tris-HCl (pH 7.5), 5 mM ZnCl2, 0.5 mM DTT, 0.5 mM EDTA, 1.5% glycerol, 100 μg/ml BSA] for 1 h at 37˚C. Sections were then subjected to immunofluorescence microscopy with anti-8-oxo-dG (1:100), without (RNase) or with HCl denaturation (RNase/HCl). MutM pretreatment significantly decreased the 8-oxo-dG IR. Scale bar = 1 mm. (**b**) Selective detection of 8-oxo-dG in nuclear or mitochondrial DNA. Immunohistochemical detection of 8-oxo-dG in cortex of 5-month-old ADH/TO-DKH mice. Upper panels: RNase-treated sections were further pretreated with 2N HCl to denature nuclear DNA, thus 8-oxo-dG in nuclear DNA was detected. Dotted nuclear 8-oxo-dG IR was detected. Lower panels: sections were pretreated with RNase to detect 8-oxo-dG in mitochondrial DNA. Diffuse cytoplasmic 8-oxo-dG IR were evident. Scale bars = 100 μm (left panels), 50 μm (middle panels), 20 μm (right panels). (**c**) Immunofluorescence detection of nuclear 8-oxo-dG in RNase/HCl treated sections. Immunofluorescent signals of 8-oxo-dG were mostly co-localized with the DAPI signal representing nuclear DNA. Scale bar = 10 μm. (**d**) Immunofluorescence detection of mitochondrial 8-oxo-dG in RNase treated sections. Immunofluorescent signals of 8-oxo-dG were co-localized with mouse TFAM (mTFAM) signals, a mitochondrial marker. Scale bar = 10 μm.

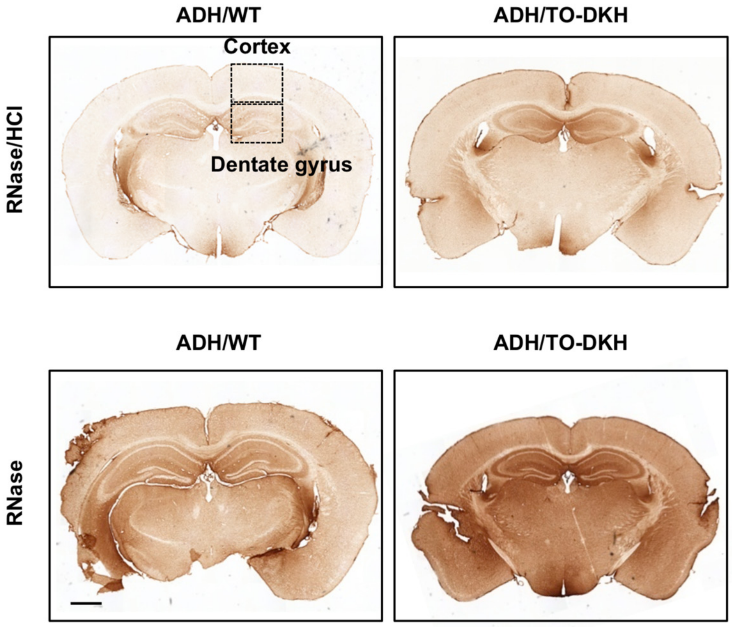

Supplementary Figure S5. Quantitative analyses of 8-oxo-dG immunoreactivity. The regions corresponding to the boxed regions of the cortex or dentate gyrus in the images shown in Figure 4a and b were analyzed. Immunodetection of 8-oxo-dG in the nuclear DNA (RNase/HCl) or mitochondrial DNA (RNase) in the cortex of 4 – 5-month-old NT, ADH/WT, and ADH/TO-DKH mice. Scale bar = 1 mm.

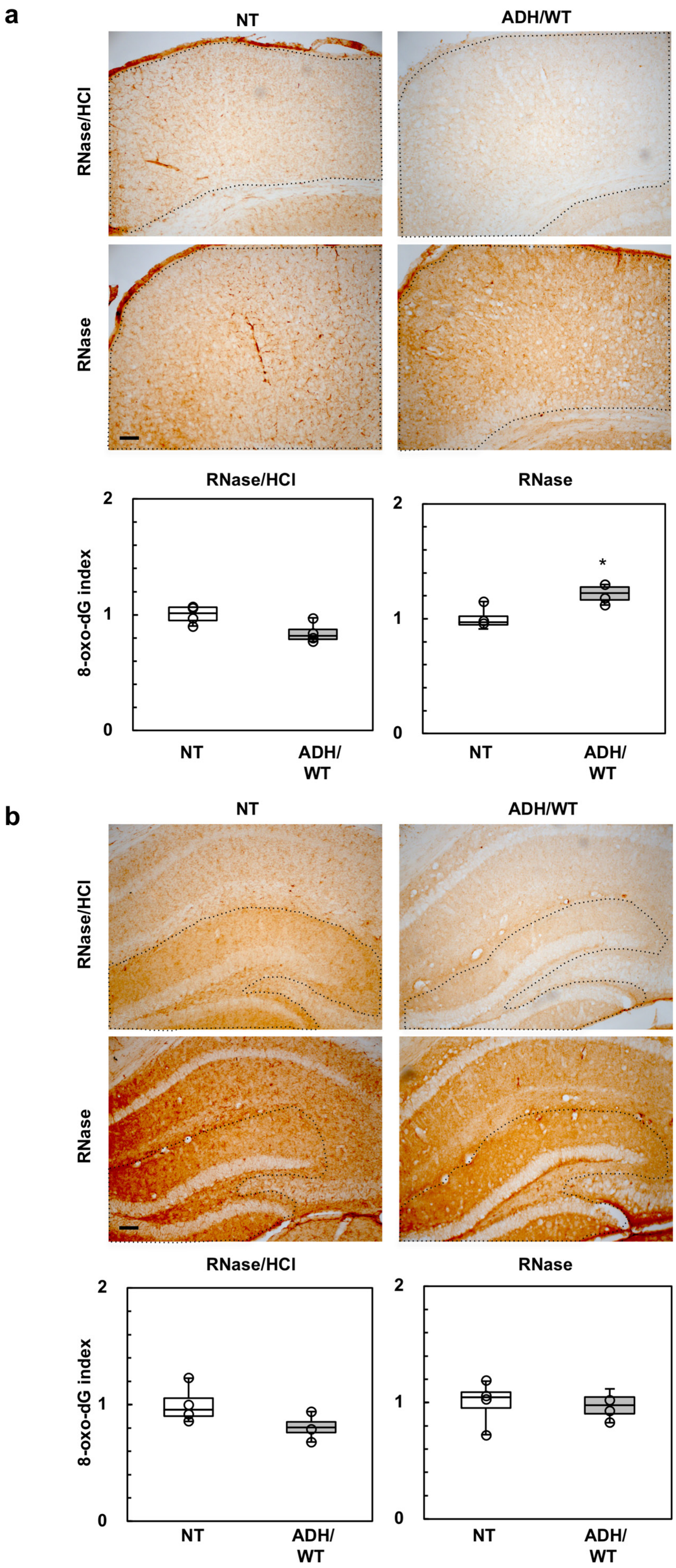
a
NT
ADH/WT
RNase/HCl
RNase
RNase/HCl
RNase
8-oxo-dG index
2
1
0
NT
ADH/
WT
*
b
NT
ADH/WT
RNase/HCl
RNase
RNase/HCl
RNase
8-oxo-dG index
2
1
0
NT
ADH/
WT

Supplementary Figure S6. No significant difference in 8-oxoG accumulation in nDNA between ADH/WT and NT brains. (**a**) Immunohistochemical detection of 8-oxo-dG in nuclei or mitochondria in the cortex of 4 – 5-month-old NT, ADH/WT mice. RNase/HCl: RNase-treated sections were further pretreated with 2N HCl to detect 8-oxo-dG in nDNA. RNase: sections were pretreated with RNase only to detect 8-oxo-dG in mtDNA. Scale bar = 100 μm. Graphs show the relative 8-oxo-dG IRs (8-oxo-dG index) in the region enclosed by the dotted line. Results from more than three mice are presented. Wilcoxon exact test (one-sided), $*p = 0.0286$. (**b**) Immunohistochemical detection of 8-oxo-dG in nuclei and mitochondria in the DG of NT, ADH/WT mice. Scale bar = 100 μm. Graphs show the relative 8-oxo-dG index in the nuclei (RNase/HCl), and mitochondria (RNase) in the DG. n = 4. Data are shown as the mean ± SEM.

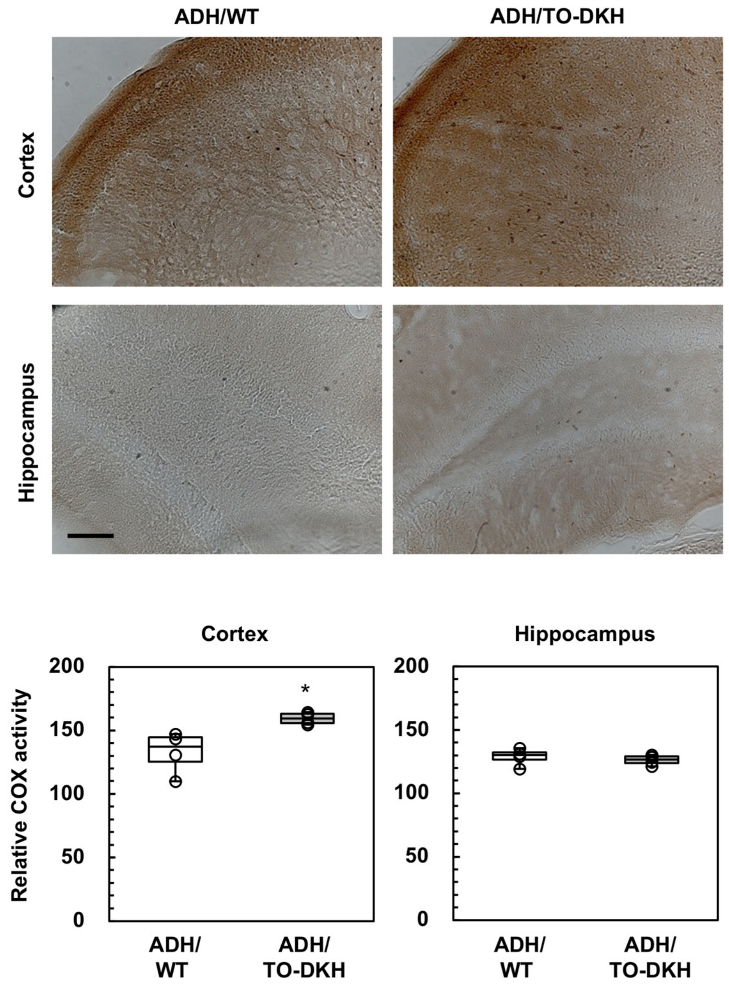

Supplementary Figure S7. *Mth1*/*Ogg1*-double knockout did not exacerbate mitochondrial dysfunction. Cytochrome c oxidase (COX) activity was not decreased in ADH/TO-DKH brain compared with ADH/WT brain. Frozen brain sections were stained for COX (brown). Scale bar, 100 µm. Relative intensity of COX staining is shown in the bar graph. n = 4. Wilcoxon exact test (two-sided), $*p = 0.0286$.

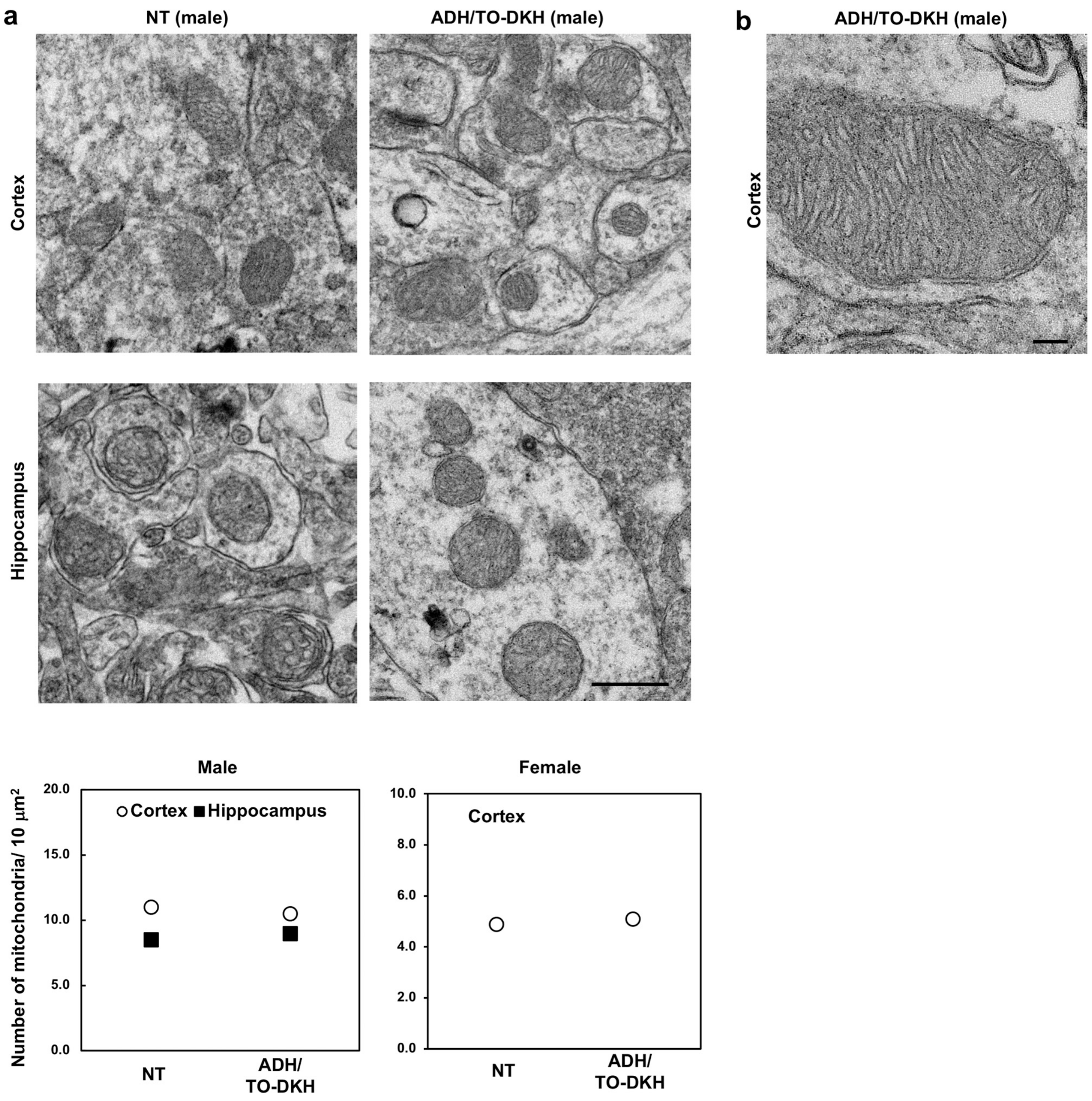

Supplementary Figure S8. Mitochondrial morphological analysis in the ADH/TO-DKH brain using transmission electron microscopy. **(a)** ADH/TO-DKH mice did not exhibit reduced mitochondrial numbers in the cortex or hippocampus compared with NT mice. Scale bar = 500 nm. Each sample was extracted from an individual mouse (male n = 1, female n = 1), different from those used for the COX assay, and 8–13 images from each mouse were analyzed. The numbers of mitochondria per 10 μm^2 are shown in the graph. (**b**) Mitochondria in the ADH/TO-DKH cortex showed intact matrices. Scale bar = 100 nm.

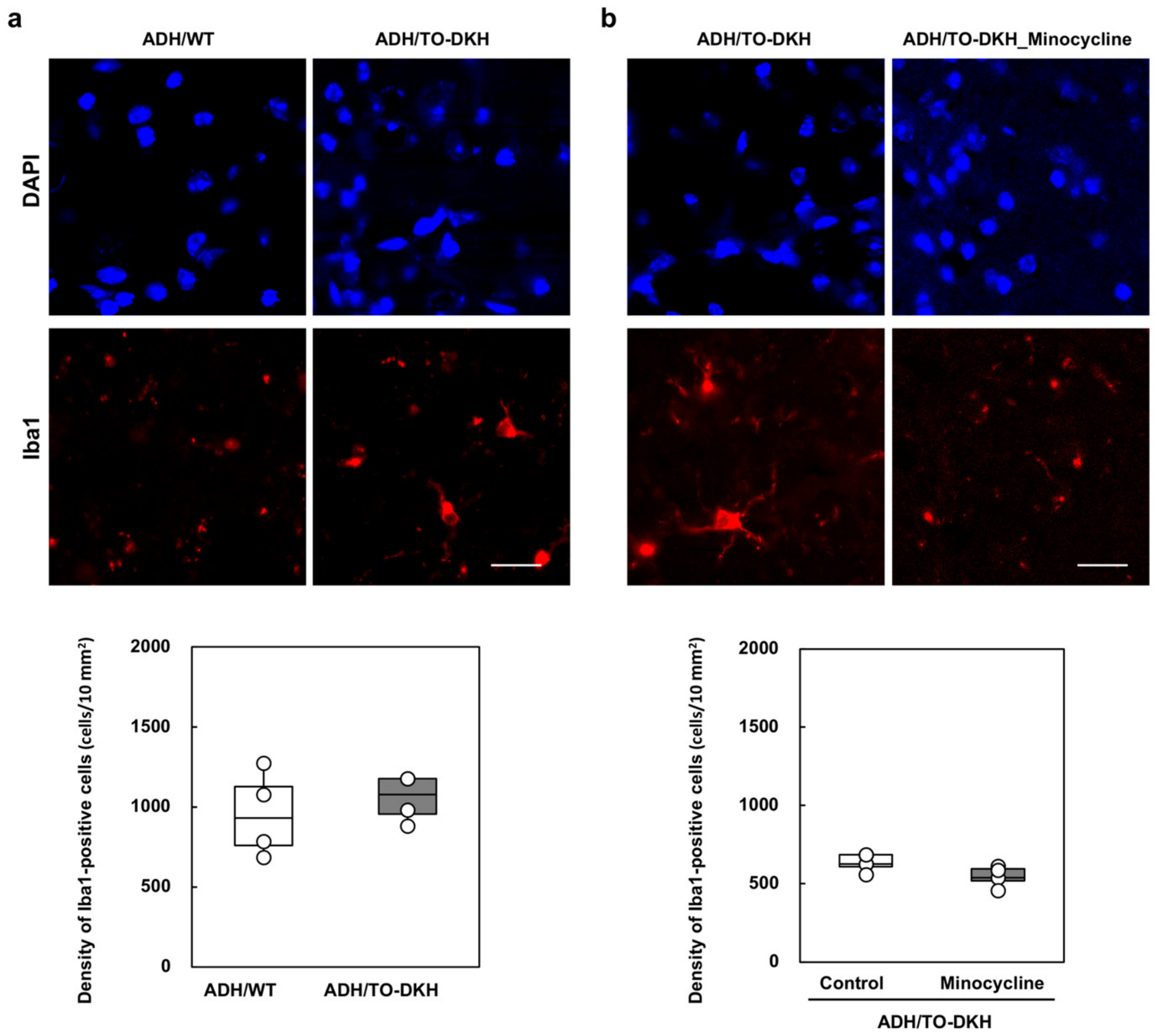

Supplementary Figure S9. Density of Iba-1-positive cells. (**a**) ADH/WT or ADH/TO-DKH cortex. Scale bar = 20 µm. n = 4. (**b**) ADH/TO-DKH cortex with or without minocycline treatment. Scale bar = 20 µm. n = 4.

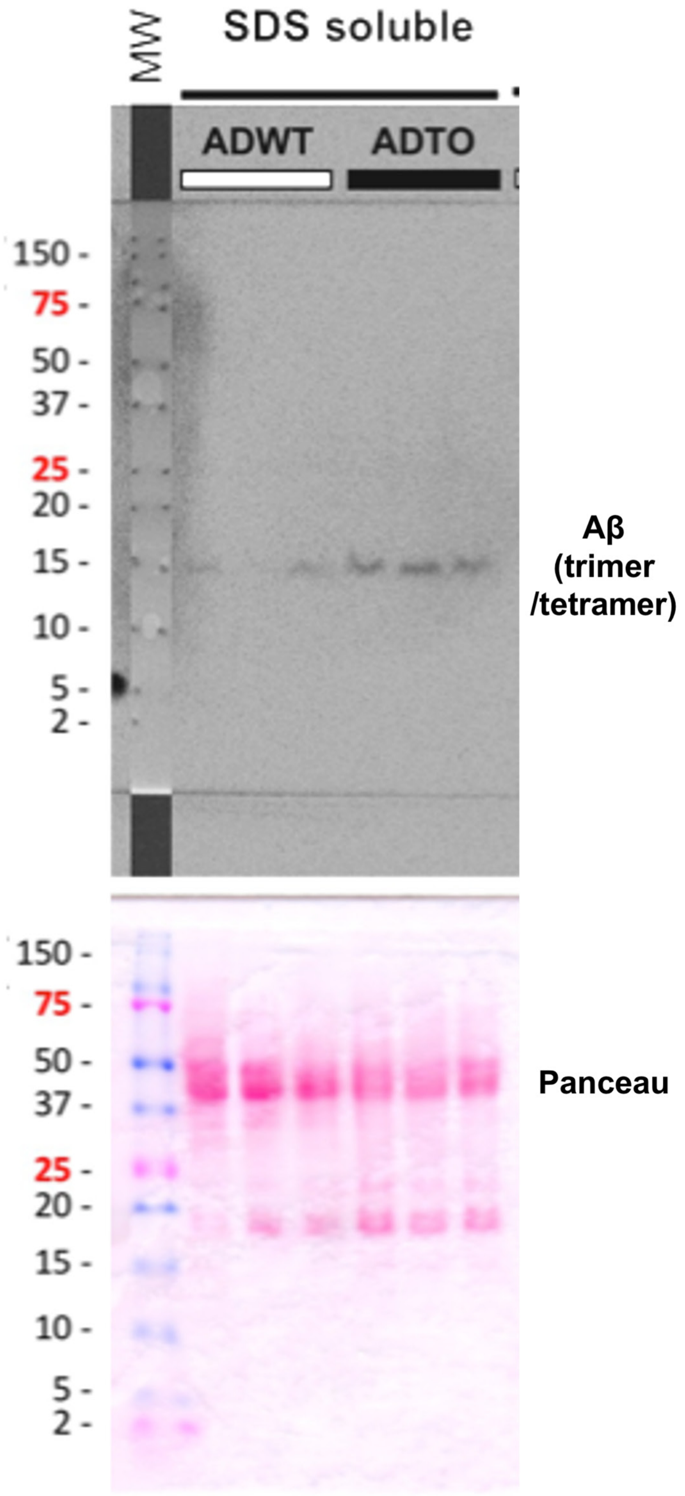

Supplementary Figure S10. The original full-length images of Figure 3c.

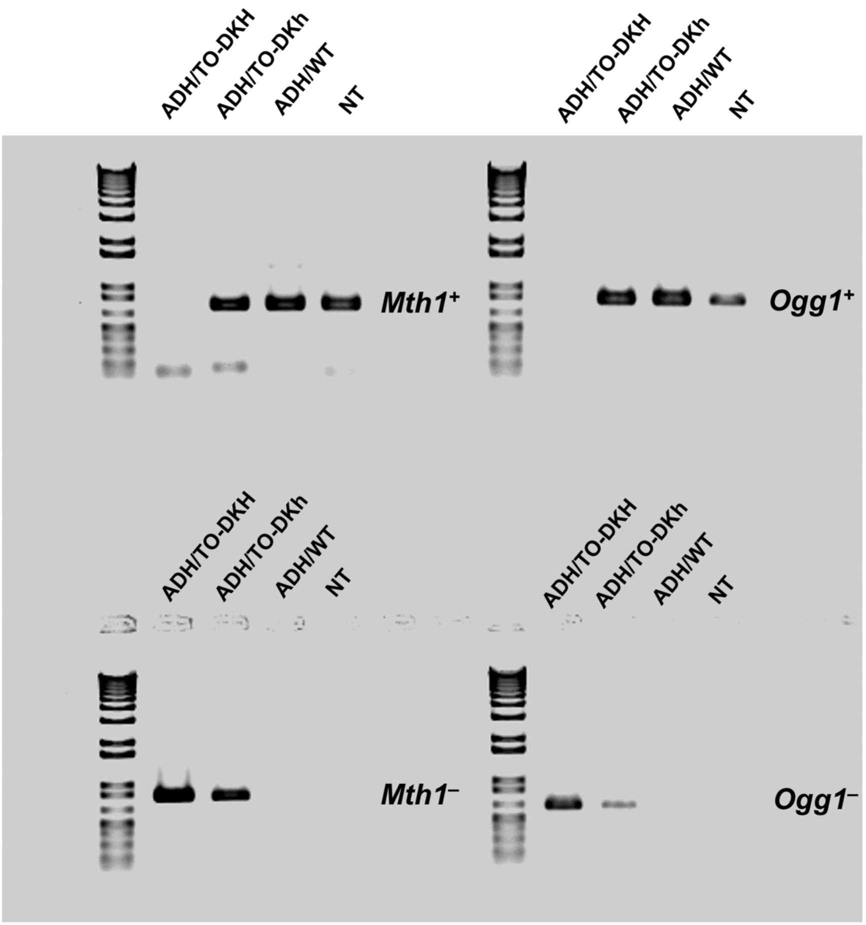

Supplementary Figure S11. The original, uncropped image of Supplementary Figure S1.
